# Single intramuscular injection of self-amplifying RNA of *Nppa* to treat myocardial infarction

**DOI:** 10.1126/science.adu9394

**Published:** 2026-03-05

**Authors:** Kaiyue Zhang, Hongyan Tao, Dashuai Zhu, Zhang Yue, Shiqi Hu, Yiping Wu, Na Yan, Yilan Hu, Shuo Liu, Mengrui Liu, Torsten Peter Vahl, Lauren Sharan Ranard, Xiao Cheng, Alexander Romanov, Jiaming Liu, Savannah Weihang Zhang, Yuan Li, Chao Lu, Ming Shen, Andrew Lewis, Ke Huang, Ke Cheng

**Affiliations:** 1 Department of Biomedical Engineering, Columbia University, New York, NY, USA.; 2 University of Oxford Centre for Clinical Magnetic Resonance Research, Radcliffe Department of Medicine, University of Oxford, Oxford, UK.; 3 Seymour, Paul and Gloria Milstein Division of Cardiology, Department of Medicine, Columbia University Irving Medical Center, New York, NY, USA.; 4 Structural Heart and Valve Center, Columbia University Irving Medical Center, New York, NY, USA.; 5 Institute of Comparative Medicine at Columbia University, New York, NY, USA.; 6 Department of Pharmaceutical Sciences, Irma Lerma Rangel College of Pharmacy, Texas A&M University, Kingsville, TX, USA.; 7 Herbert Irving Comprehensive Cancer Center, Columbia University, New York, NY, USA.

## Abstract

Self-amplifying RNA (saRNA) enables sustained protein expression from a single administration. In this study, we developed an intramuscular saRNA-lipid nanoparticle (saNppa-LNP) therapy encoding natriuretic peptide type A (*Nppa*) for cardioprotection. A single injection induced sustained pro–atrial natriuretic peptide (pro-ANP) secretion for 4 weeks; pro-ANP was subsequently cleaved by the cardiac protease corin into active ANP, producing robust cardioprotection in mouse and swine myocardial infarction models. At equivalent doses, saNppa achieved greater efficacy than conventional mRNA. Single-nucleus transcriptomics identified natriuretic peptide receptor 1–positive (*Npr1*^+^) endothelial and epicardial cells as primary effectors, with saNppa-LNPs reshaping their paracrine profile to promote cardiomyocyte regeneration and suppress fibrosis. Longitudinal biosafety assessments revealed no systemic toxicity. Together, these results demonstrate that one-shot saNppa-LNP therapy offers durable cardioprotection, supporting the broader potential of saRNA-LNP–based approaches for cardiac therapy.

## Abstract (summary)

**INTRODUCTION:**

Myocardial infarction (MI) causes irreversible loss of cardiomyocytes and adverse remodeling, ultimately progressing to heart failure. Although gene- and RNA-based therapies offer promising strategies for cardiac repair, current approaches often rely on invasive myocardial delivery or are limited by short expression duration and low protein yields of conventional mRNA. Therefore, the development of a minimally invasive therapeutic platform that enables durable expression of cardioprotective factors remains a critical unmet need in cardiac therapy.

**RATIONALE:**

Atrial natriuretic peptide (ANP), encoded by natriuretic peptide type A (*Nppa*), is a developmentally regulated cardiac hormone with promising cardioprotective functions. We observed that *Nppa* expression was induced in both neonatal and adult mouse hearts after MI; however, the magnitude of induction was markedly greater in regenerative neonatal hearts. This disparity suggested that higher levels of *Nppa* expression may be associated with enhanced regenerative capacity and that insufficient *Nppa* induction may limit cardiac repair in adults. To address this, we developed a self-amplifying RNA (saRNA) therapy delivered via lipid nanoparticles (LNPs) to drive additional *Nppa* expression. Unlike mRNA, saRNA enables intracellular RNA replication, resulting in prolonged protein expression at substantially lower doses. We hypothesized that a single intramuscular (IM) injection of an saRNA-LNP encoding native *Nppa* (saNppa-LNP) could establish an in vivo “RNA factory,” continuously producing circulating pro-ANP that is selectively activated into functional ANP in the heart by the cardiac protease corin, thereby providing durable cardioprotection without direct cardiac manipulation.

**RESULTS:**

A single IM injection of saNppa-LNPs in mice induced robust pro-ANP secretion lasting at least 4 weeks, outperforming mRNA at equivalent doses. In mouse models of acute MI and ischemia/reperfusion (I/R) injury, saNppa-LNP treatment markedly improved left ventricular ejection fraction, reduced infarct size, and attenuated fibrosis. These therapeutic benefits were consistently reproduced in aged, atherosclerotic, and diabetic MI models. In addition, large-animal studies in a swine I/R model further confirmed that a single IM injection effectively preserved cardiac function and limited maladaptive remodeling. Mechanistically, single-nucleus transcriptomics revealed that saNppa-LNP treatment reshaped the paracrine profile of natriuretic peptide receptor 1–positive (*Npr1*^+^) endothelial and epicardial cells, creating a proregenerative microenvironment that promoted cardiomyocyte cell-cycle reentry and suppressed the expansion of profibrotic periostin-positive (*Postn*^+^) fibroblasts. Longitudinal safety assessments revealed only transient local inflammation, with no evidence of adaptive immune activation or systemic toxicity.

**CONCLUSION:**

Our study demonstrates that a single IM administration of saNppa-LNPs provides robust and durable cardioprotection across multiple species and clinically relevant injury models. By leveraging the self-amplifying properties of saRNA and the heart-specific activation of pro-ANP, this minimally invasive, one-shot therapy may offer a safe, simple, and effective strategy for cardiac repair. □

## Graphical Abstract

Intramuscular injectable saNppa-LNP therapy for durable cardioprotection.

A single IM injection of saNppa-LNPs enables self-amplification and high-yield pro-ANP production for more than 28 days. Circulating pro-ANP is selectively cleaved by the cardiac protease corin into active ANP, activating NPR1/cGMP signaling in the heart. At equivalent doses, this minimally invasive, longacting approach confers greater cardioprotection efficacy compared with conventional mRNA. GTP, guanosine triphosphate; cGMP, cyclic guanosine 3′,5′-monophosphate; UTR, untranslated region. [Figure created with BioRender.com]

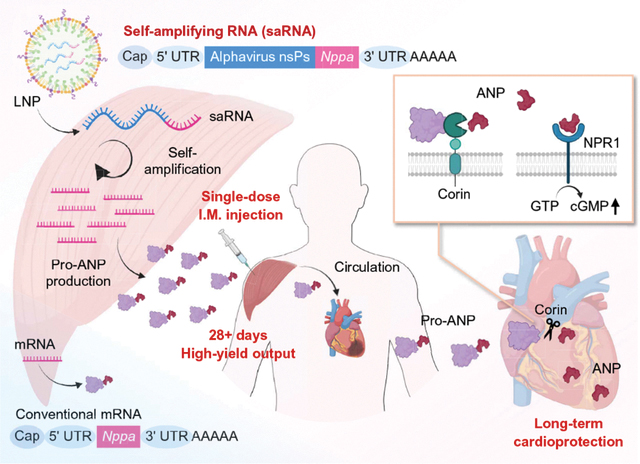

In the adult mammalian heart, myocardial infarction (MI) invariably leads to myocardial damage and fibrosis (1, 2). Therapeutic reperfusion by percutaneous coronary intervention immediately after MI reduces the infarcted area but also contributes to further injury through sudden oxidative stress (3). Emerging gene- and RNA-based therapies aim to restore cardiac function by expressing protective molecules in the heart, which typically require intramyocardial injections or targeted delivery (4–7). However, most of these approaches face notable challenges: Intramyocardial injections usually require complex procedures, such as open-chest direct injection or catheter-based procedures, which place high demands on personnel and facilities; in addition, targeted delivery to the heart is extremely difficult, and off-target effects remain unavoidable (7–10). Therefore, alternative strategies have been explored to express circulating cardioprotective factors distally, allowing secretion of therapeutic molecules that act on the heart. For example, adeno-associated virus (AAV)–mediated hepatic expression of circulating cardioprotective proteins has been shown to promote post-MI repair (11). It is highly desirable to have drugs that can be delivered through simple and minimally invasive routes, such as intravenous (IV) or intramuscular (IM) injections, and ultimately exert therapeutic effects in the heart.

RNA-lipid nanoparticle (LNP) technology, which has been successfully applied in COVID-19 vaccine development (12–16), represents a next-generation delivery strategy that may offer an alternative to AAV for delivering circulating cardioprotective factors (17–20). However, the utility of conventional linear mRNA technology is limited by inherent deficiencies, including low protein yield and short expression duration, which compel the development of long-acting RNA platforms (21–23). Self-amplifying RNA (saRNA), derived from positive-sense RNA viruses such as alphaviruses, is an engineered RNA system that encodes both the protein of interest and a viral replicase, enabling intracellular self-replication and sustained, high-level protein expression from a small dose (24–30). Such an approach offers the potential to generate sufficient circulating cardioactive factors at a safe RNA-LNP dose, thereby offering a promising approach for cardiac therapy.

Atrial natriuretic peptide (ANP), encoded by the natriuretic peptide type A (*Nppa*) gene, is a cardiac hormone that is secreted predominantly by the atria and plays essential roles in cardiovascular homeostasis (31–33). During development, *Nppa* is highly expressed in the fetal atria and the trabeculated ventricular myocardium but is largely silenced in the postnatal ventricles (34). *Nppa* expression is up-regulated in the infarct border zone after MI (34). The reengagement of the fetal gene program suggests that ANP regulates cardiac development and regeneration in a spatiotemporal manner. Previous studies have reported multiple cardioprotective effects of ANP, including anti-hypertrophic, proangiogenic, and antifibrotic functions, and therapeutic applications of natriuretic peptides have been investigated in experimental and clinical settings (35–37). Reanalysis of a previously published sequencing dataset suggested that *Nppa* expression is more strongly induced in neonatal hearts than in adult hearts after MI (38), which is consistent with the greater regenerative potential of neonatal myocardium (39, 40). This association suggests that ANP may contribute to regenerative repair and highlights its potential as a circulating cardioprotective factor for therapeutic exploration. Here, we developed an intramuscularly injectable long-acting RNA therapy that harnesses saRNA and LNP technology to achieve sustained ANP production, offering a promising strategy for durable cardioprotection after MI (Fig. 1A).

## Pro-ANP was overexpressed in neonatal hearts after MI and promoted cardiac repair

**Fig. 1.**
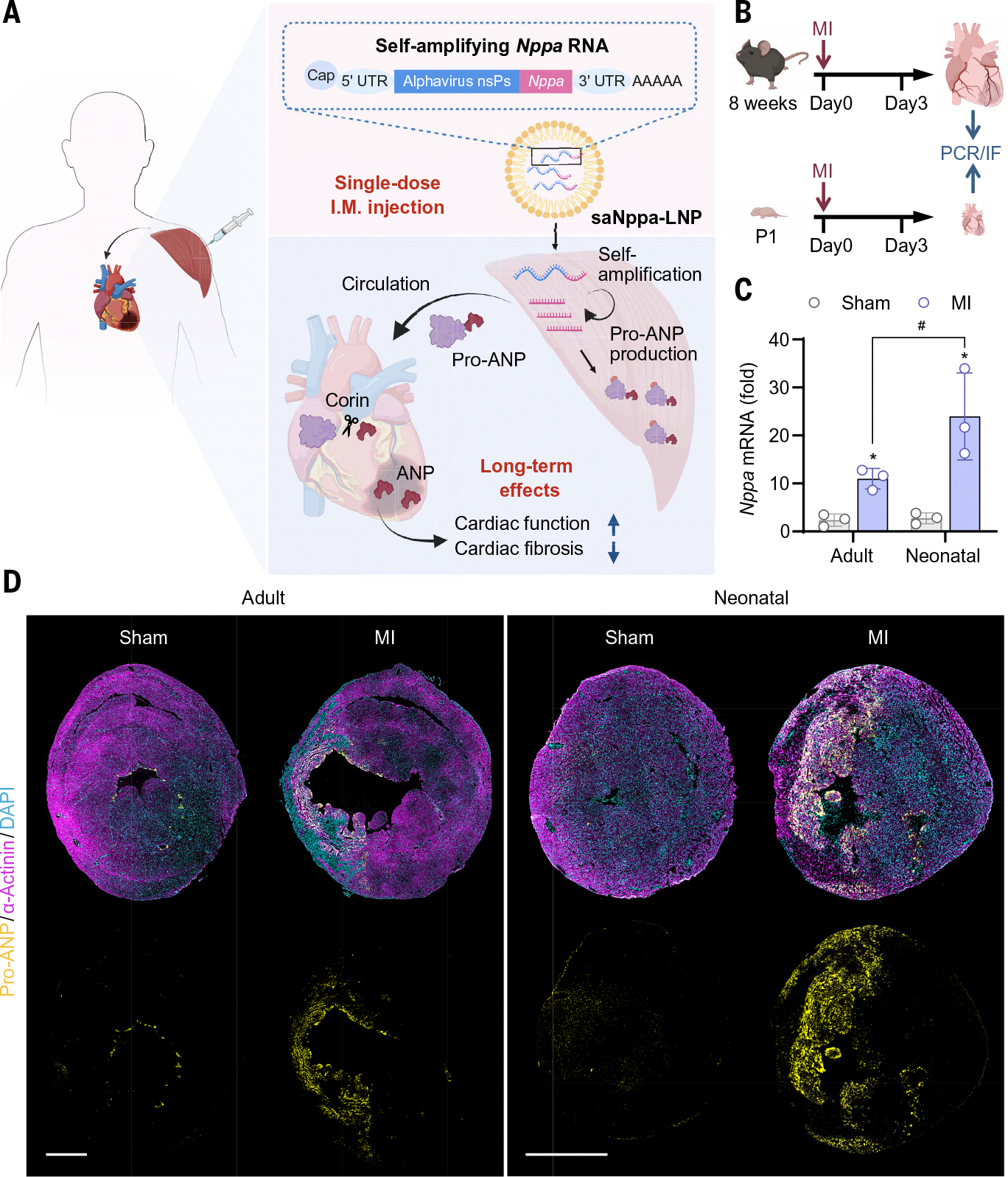
Cardioprotective factor pro-ANP expression was insufficient in adult hearts after MI. (**A**) Schematic showing that intramuscularly injected saNppa-LNPs promote heart repair after MI. saRNA encoding the *Nppa* gene were packaged into LNPs and then injected into muscle tissue, where the *Nppa* mRNA was amplified and translated to produce pro-ANP. Pro-ANP was released into the circulation and subsequently cleaved into functional ANP by corin in the heart, ultimately achieving cardioprotective effects by protecting cardiac function and inhibiting fibrosis. UTR, untranslated region. [Figure created with BioRender.com] (**B**) Schematic of the experimental design showing collection of border-zone tissues from neonatal and adult mouse hearts on day 3 after MI. (**C**) *Nppa* mRNA levels in the border zone of adult and neonatal hearts on day 3 after MI. All data were normalized to samples collected from sham-operated adult mice. Data are expressed as mean ± SD. *n* = 3. Statistical analysis was performed using one-way ANOVA with Tukey’s multiple comparison test. *P < 0.05 versus age-matched sham-operated group, ^#^*P* < 0.05 between the indicated groups. (**D**) mmunofluorescence staining of pro-ANP (yellow) in hearts from sham-operated and MI mice (α-actinin, magenta, CMs) on day 3 after MI. Nuclei were counterstained with 4′,6-diamidino-2-phenylindole (DAPI) (cyan). Scale bars, 1 mm.

To experimentally test whether *Nppa* expression is more strongly induced in neonatal than in adult hearts after MI, we collected border zone left ventricular (LV) tissues from mouse neonatal and adult hearts at day 3 after MI (Fig. 1B). Real-time polymerase chain reaction (PCR) revealed that MI induced around 10-fold *Nppa* up-regulation in adult hearts but more than 25-fold in neonatal hearts (Fig. 1C). Immunofluorescence (IF) staining of pro-ANP (in yellow) confirmed this difference: Pro-ANP was rarely detectable in hearts from sham-operated adult or neonatal mice, whereas after MI, pro-ANP expression was observed in the MI border region of both adult and neonatal hearts, with markedly higher levels in neonatal hearts (Fig. 1D). To further test its functional role, we knocked down endogenous *Nppa* expression in neonatal mouse hearts subjected to MI, using an AAV9–short hairpin RNA (AAV9-shRNA) cocktail, and assessed outcomes on day 28 (fig. S1). The knockdown efficiency of the AAV9-shRNA cocktail was validated by real-time PCR and IF staining on day 7 (fig. S1, A to C). Echocardiography revealed that *Nppa* knockdown significantly reduced LV ejection fraction (LVEF) from ~60% to <40% (*P* = 0.003) (fig. S1, D to F). Consistently, Masson’s trichrome staining revealed extensive fibrosis and adverse remodeling in the *Nppa*-knockdown group compared with the MI control or the MI plus AAV–negative control (AAV-NC) injection group (fig. S1, G and H), suggesting that *Nppa* is needed for neonatal heart regeneration. Taken together, these findings suggest that *Nppa*-encoded pro-ANP is a critical positive regulator of neonatal cardiac repair, whereas its expression in the adult heart is insufficient.

## Intramuscular injection of saNppa-LNPs sustained pro-ANP production and activated cardiac ANP signaling

**Fig. 2.**
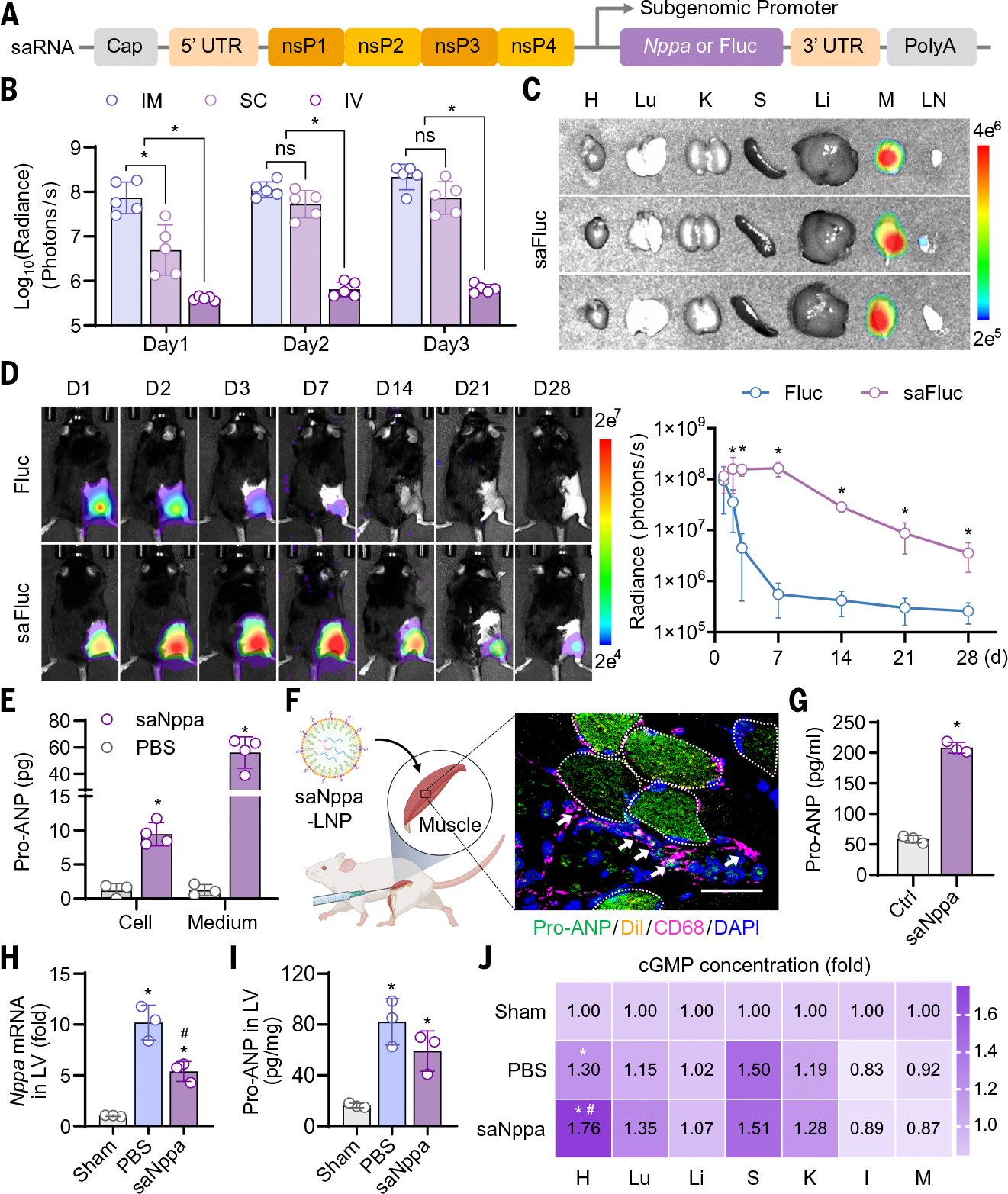
Fabrication and characterization of an intramuscularly injectable saNppa-LNP. (**A**) Schematic illustrating the structure of saNppa RNA. (**B**) Bioluminescence radiances of mice receiving a single IM, SC, or IV injection of 5 μg saFluc-LNPs, measured on day 1, 2, and 3 after injection. Radiance of the Fluc signal is expressed as photons/s. Data are presented as mean ± SD. *n* = 5. Significance was determined by one-way ANOVA with Tukey’s multiple comparisons test. *P < 0.05 between the indicated groups. ns, not significant. (**C**) Organ distribution of saFluc-LNPs in mice shown by Fluc radiance on day 3 after a single IM injection of 5 μg saFluc-LNPs. H, heart; Lu, lung; K, kidney; S, spleen; Li, liver; M, muscle; LN, lymph node. (**D**) Duration of Fluc expression after a single IM injection of 5 μg saFluc-LNPs was traced in vivo by longitudinal Fluc imaging. Radiance of the Fluc signal is expressed as photons/s. Data are presented as mean ± SD. *n* = 5. Significance was determined by two-way ANOVA with Tukey’s multiple comparison test. *P < 0.05 versus Fluc. (**E**) Pro-ANP protein levels in HEK 293 cell lysates and culture medium at 24 hours after treatment with 100 ng saNppa-LNPs, measured by ELISA. Data are presented as mean ± SD. *n* = 3 or 4. Significance was determined by two-way ANOVA with Tukey’s multiple comparison test. *P < 0.05 versus PBS. (**F**) Schematic illustrating that saNppa-LNPs were intramuscularly injected into the mouse hindlimb. [Figure created with BioRender.com] Confocal micrograph of pro-ANP (green), LNPs (DiI, yellow), and CD68 (magenta) in a muscle tissue section on day 3 after saNppa-LNP injection. Nuclei were counterstained with DAPI (blue). Scale bar, 50 μm. Dashed circles outline pro-ANP–expressing skeletal muscle fibers, and arrows indicate pro-ANP–expressing macrophages. (**G**) Pro-ANP levels in serum of normal mice on day 3 after IM injection of saNppa-LNPs. An equal volume of PBS was injected as control. Data are presented as mean ± SD. *n* = 3. Statistical analysis was performed using a two-tailed unpaired Student’s *t*-test. *P < 0.05 versus control (Ctrl). (**H**) *Nppa* mRNA and (**I**) pro-ANP protein levels in LV tissues on day 3 after MI and 5 μg saNppa-LNP injection. Data are presented as mean ± SD. *n* = 3. Significance was determined by one-way ANOVA with Tukey’s multiple comparison test. *P < 0.05 versus Sham, ^#^*P* < 0.05 versus PBS. (**J**) cGMP fold changes in hearts and other main organs on day 3 after MI and saNppa-LNP injection. H, heart; Lu, lung; Li, liver; S, spleen; K, kidney; I, intestine; M, muscle. Data are normalized to organs collected from sham-operated mice and presented as fold change. *n* = 3. Significance was determined by two-way ANOVA with Tukey’s multiple comparison test. *P < 0.05 versus Sham, ^#^*P* < 0.05 versus PBS.

To compensate for the insufficient *Nppa* expression in injured adult hearts, we designed an RNA-LNP system to express additional pro-ANP in vivo. Given the short half-life of ANP in circulation, typically only a few minutes (41), we constructed a self-amplifying *Nppa* (saNppa) RNA plasmid template for in vitro transcription (IVT) and synthesized capped and polyadenylated saNppa RNA (fig. S2A). Unlike conventional mRNA, saNppa RNA encodes four nonstructural proteins (nsPs) that enable intracellular replication of the *Nppa* transcript, thereby sustaining its expression (Fig. 2A). To identify an optimal delivery system, we screened three Food and Drug Administration (FDA)–approved LNP formulations (DLin-MC3-DMA, ALC-0315, and SM-102) (fig. S2B). Characterization of particle size and ζ potential of saNppa-LNPs—together with assessment of transfection and expression efficiency obtained using self-amplifying green fluorescent protein (saGFP) reporter RNA—revealed that SM-102, the cationic lipid formulation used in the Moderna COVID-19 vaccine, provided the most effective delivery of saRNA (fig. S2, C to F). Therefore, SM-102–based LNPs were selected for subsequent experiments. Characterization of the final saNppa-LNP product showed the RNA loading efficiency exceeding 90% and well-defined morphology by cryo–electron microscopy (cryo-EM) (fig. S2, G and H).

To determine the optimal administration route for saRNA-LNPs, we compared IV, subcutaneous (SC), and IM injections in mice, using self-amplifying firefly luciferase (saFluc) reporter RNA. After injection of 5 μg saFluc-LNPs, both SC and IM injections produced robust bioluminescent signals, whereas IV injection failed to yield detectable expression. IM injection generated stronger signals than SC on day 1, and therefore we selected IM injection for saRNA-LNP delivery in our study (Fig. 2B and fig. S3A). Subsequently, we examined the tissue distribution and duration of saRNA-LNP expression after IM injection. Bioluminescence imaging of mice and organs from animals injected intramuscularly with 5 μg saFluc-LNPs showed that Fluc expression was strictly confined to the injected hindlimb muscles, with no detectable leakage to major organs such as the livers or spleens (Fig. 2C and fig. S3, B to D). To further characterize expression persistence, we compared conventional Fluc and GFP mRNA with saFluc in vivo and saGFP in vitro. Conventional Fluc mRNA expression diminished within 1 week, whereas a single IM injection of saFluc-LNPs sustained robust luciferase expression in mouse hindlimbs for at least 4 weeks (Fig. 2D). Consistently, human embryonic kidney (HEK) 293 cells transfected with saGFP-LNPs also exhibited stable expression for more than 4 weeks (fig. S3, E and F).

To confirm functional expression of saNppa, we transfected HEK 293 cells with saNppa-LNPs and detected pro-ANP in both cell lysates and culture medium (Fig. 2E and fig. S4, A and B), demonstrating that the fabricated saNppa-LNPs effectively produced and secreted exogenous pro-ANP. In vivo and ex vivo organ distribution analysis of DiR-labeled saNppa-LNPs revealed a pattern similar to that observed with saFluc-LNPs after IM injection in mice (fig. S4, C to E). Furthermore, IF staining of injected muscle tissues showed that saNppa-LNPs were taken up and expressed in both muscle fibers and infiltrating macrophages at the injection sites (Fig. 2F and fig. S5).

Subsequently, we investigated whether exogenously produced pro-ANP from IM injection of saNppa-LNPs could function in the injured heart. After IM injection of 5 μg saNppa-LNPs into healthy mice, the observed elevation of serum pro-ANP confirmed that exogenous pro-ANP produced by muscle tissue went into the circulation (Fig. 2G). Because pro-ANP requires cleavage by the transmembrane proteinase corin to generate biologically active ANP (31, 42), we examined corin distribution across organs in mice (fig. S6, A to C). Western blot and single-nucleus RNA sequencing (snRNA-seq) analysis revealed that corin expression was strongly enriched in the heart, especially in cardiomyocytes (CMs) (fig. S6, D to F), which is consistent with prior human data (43). To assess cardiac responsiveness to exogenous pro-ANP, we measured *Nppa* mRNA, pro-ANP protein, and its downstream effector cyclic guanosine 3′,5′-monophosphate (cGMP) in LV tissues after MI and 5 μg saNppa-LNP injection. Endogenous *Nppa* mRNA expression was significantly down-regulated after injection (*P* = 0.0048) (Fig. 2H), suggesting that sufficient replenishment of exogenous pro-ANP relieved cardiac stress and triggered a negative feedback response. Total pro-ANP protein levels in LV tissue did not show a significant reduction compared with the phosphate-buffered saline (PBS) control group (Fig. 2I). This is likely because our assay primarily detected the decreased intracellular pro-ANP within CMs, whereas circulating exogenous pro-ANP was lost during PBS perfusion before tissue collection. To ensure that exogenous pro-ANP remains under endogenous regulatory control, we expressed the native, tag-free form of pro-ANP; however, this precluded direct detection of the exogenous pro-ANP, so we instead assessed its functional activation by measuring downstream effectors in the ANP/NPR1/cGMP pathway (NPR1, natriuretic peptide receptor 1). cGMP levels were significantly increased in LV tissue after saNppa-LNP injection (*P* = 0.0051), whereas no significant changes were observed in lung, kidney, and other organs (Fig. 2J). Together, these findings demonstrate that IM injection of saNppa-LNPs generates native, secretion-competent pro-ANP that circulates systemically, is specifically activated in the heart by corin, and effectively stimulates the ANP/NPR1/cGMP signaling pathway in the injured heart.

## Single IM injection of saNppa-LNPs promoted heart repair in mice with acute MI

To evaluate the therapeutic efficacy of a single dose of saNppa-LNPs, we established a mouse model of acute MI by ligation of the left anterior descending artery (LAD). Dose optimization was performed by IM injection of 0.05 mg/kg, 0.25 mg/kg, or 0.5 mg/kg saNppa-LNPs. Echocardiography and Masson’s trichrome staining on day 28 revealed that all doses notably improved cardiac function and structure compared with PBS, but 0.25 mg/kg and 0.5 mg/kg saNppa-LNPs produced comparable benefits, superior to those produced by the 0.05 mg/kg group (fig. S7, A to D). On the basis of these findings, 0.25 mg/kg was selected for subsequent studies.

**Fig. 3.**
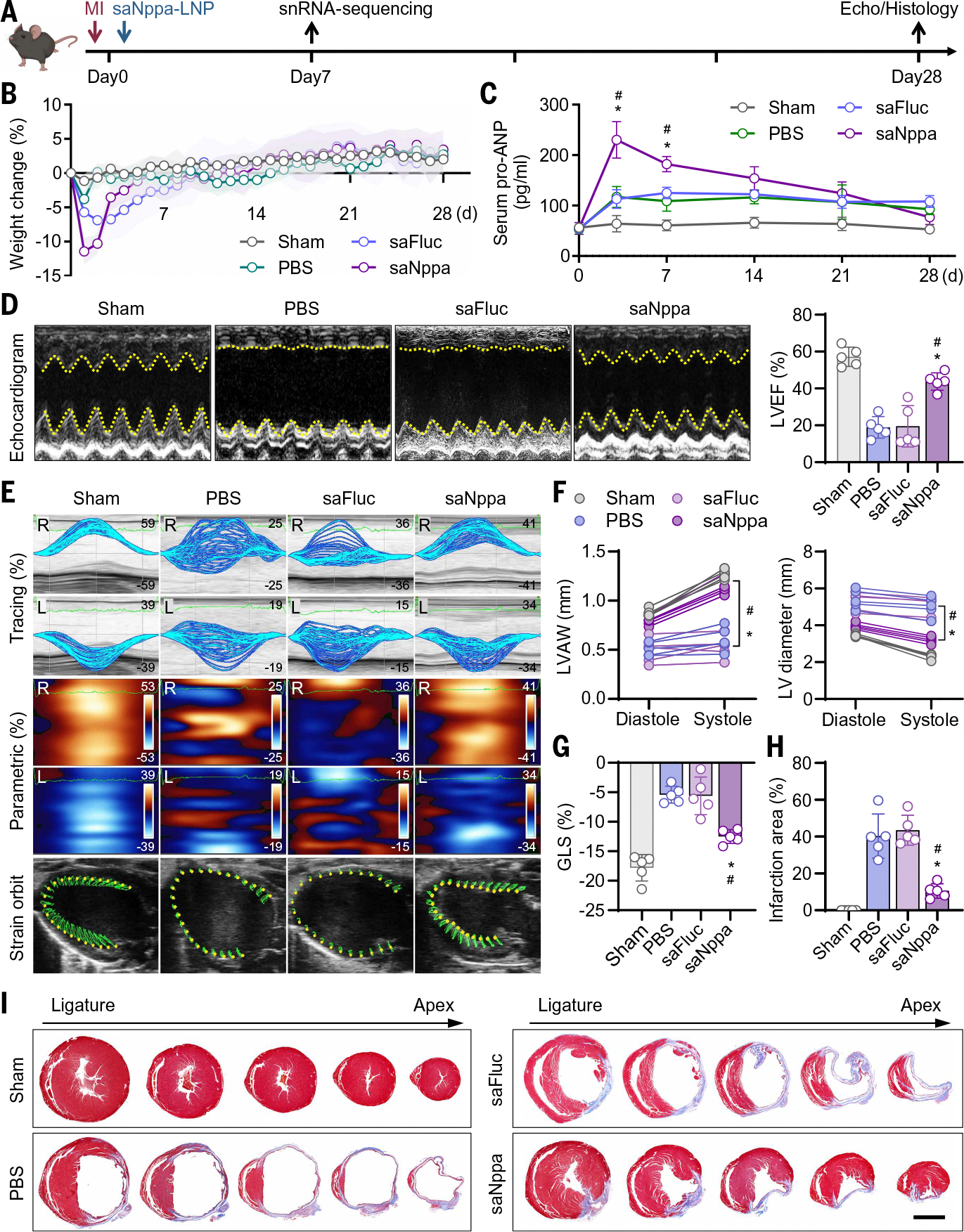
Cardioprotection by a single IM injection of saNppa-LNP. (**A**) Schematic showing animal study design. (**B**) Body weight changes of female mice after MI and treatment. Data are presented as mean ± SD. *n* = 3. (**C**) Longitudinal analysis of pro-ANP levels in mouse serum after injection of 0.25 mg/kg saNppa-LNPs. Data are presented as mean ± SD. *n* = 3. Statistical analysis was performed using two-way ANOVA with Tukey’s multiple comparison test. *P < 0.05 versus PBS, ^#^*P* < 0.05 versus saFluc. (**D**) Representative echocardiography images and quantification of LV ejection infarction (LVEF). Data are presented as mean ± SD. *n* = 5. Statistical analysis was performed using one-way ANOVA with Tukey’s multiple comparison test. *P < 0.05 versus PBS, ^#^*P* < 0.05 versus saFluc. (**E**) Representative strain tracings (top) and parametric distribution maps (middle) of the LV endocardium along radial and longitudinal axes. Strain tracings (*y* axis) for each speckle are displayed along the traced contour over time (*x* axis). For radial parametric distribution maps, movement of each speckle is displayed as blue (moving away from the center of the heart) or red (moving toward the center of the heart) over time (*x* axis). For longitudinal parametric distribution maps, blue indicates tissue moving away from the apex, and red indicates tissue moving toward the apex. Strain orbit images (bottom) show the LV endocardium in B-mode long-axis views with tracked endocardial points. (**F**) Measurements of LV anterolateral wall (LVAW) thickness and LV diameter. Data are presented as individual values. *n* = 5. Statistical analysis was performed using two-way ANOVA with Tukey’s multiple comparison test. *P < 0.05 versus PBS, ^#^*P* < 0.05 versus saFluc. (**G**) Quantification of LV endocardium global longitudinal strain (GLS). Data are presented as mean ± SD. *n* = 5. Statistical analysis was performed using one-way ANOVA with Tukey’s multiple comparison test. *P < 0.05 versus PBS, ^#^*P* < 0.05 versus saFluc. (**H**) Measurements of infarction area at the ligature site by Masson’s trichrome staining. Data are presented as mean ± SD. *n* = 5. Statistical analysis was performed using one-way ANOVA with Tukey’s multiple comparison test. *P < 0.05 versus PBS, ^#^*P* < 0.05 versus saFluc. (**I**) Representative Masson’s trichrome staining images of sequential heart cross sections from the ligature site to the apex. Scale bar, 2 mm.

Next, we assessed the cardioprotective effects of 0.25 mg/kg saNppa-LNPs in both female (Fig. 3) and male (fig. S8) MI mice. Mice underwent LAD ligation followed by IM injection of saNppa-LNPs on the same day, with saFluc-LNPs or PBS administered as controls. Cardiac function and remodeling were evaluated on day 28 after MI by echocardiography and histology (Fig. 3A and fig. S8A). During the 4-week period, body weight and serum samples were collected for longitudinal monitoring. Body weight analysis revealed that saRNA-LNP injections caused transient weight loss during the first week compared with non–saRNA-LNP injected groups. Mice after saNppa-LNP injection lost up to 10% of body weight in the first 3 days, compared with ~7% in the saFluc-LNP group, which is consistent with the known systemic natriuretic and diuretic effects of ANP (Fig. 3B and fig. S8B). This early response to exogenous ANP likely contributed to reducing cardiac stress during the initial stage of MI recovery. Serum pro-ANP levels increased approximately twofold (~100 pg/ml) after MI compared with the sham-operated group (~50 pg/ml), and saNppa-LNP injection further raised the level to ~200 pg/ml at day 3 after injection. Thereafter, pro-ANP levels declined to baseline within 3 weeks in the saNppa-LNP group, whereas levels remained persistently elevated (~100 pg/ml) in the PBS and saFluc-LNP groups (Fig. 3C). These dynamics suggest that although endogenous pro-ANP was induced by MI, the elevation was insufficient to preserve cardiac function, which led to a continuous injury stimulation to elevate pro-ANP level. By supplementing exogenous pro-ANP, saNppa-LNPs accelerated repair, allowing serum levels back to normal by day 28.

Echocardiography on day 28 demonstrated that a single IM injection of saNppa-LNPs markedly improved LV performance, with a LVEF of 40% in saNppa-LNP–treated hearts as compared with ~20% in mice that received placebos (PBS or saFluc) (Fig. 3D and fig. S8, C and D). Treatment also restored LV structure, with thickening of the infarcted LV anterolateral wall (LVAW) and reduced LV chamber dilatation (Fig. 3F and fig. S8D). Myocardial strain analysis provided further evidence of functional recovery: Strain tracing and parametric maps, as well as strain orbit, revealed that saNppa-LNPs ameliorated MI-induced desynchronization of myocardial deformation in both radial and longitudinal axes (Fig. 3E and fig. S8, E and F). Quantification of global longitudinal strain (GLS) showed a significant improvement after saNppa-LNP treatment (*P* = 0.0005 in female mice and *P* = 0.0032 in male mice) (Fig. 3G and fig. S8G). Consistent with these findings, Masson’s trichrome staining of whole-heart sections confirmed that saNppa-LNPs reduced infarct size, attenuated fibrosis, and limited maladaptive remodeling (Fig. 3, H and I, and fig. S8, H and I). These therapeutic benefits were uniformly seen in both sexes of mice (fig. S9).

## saNppa-LNPs promoted cardiac recovery and reduced fibrosis by reshaping the paracrine profile of *Npr1*^+^ cells

**Fig. 4.**
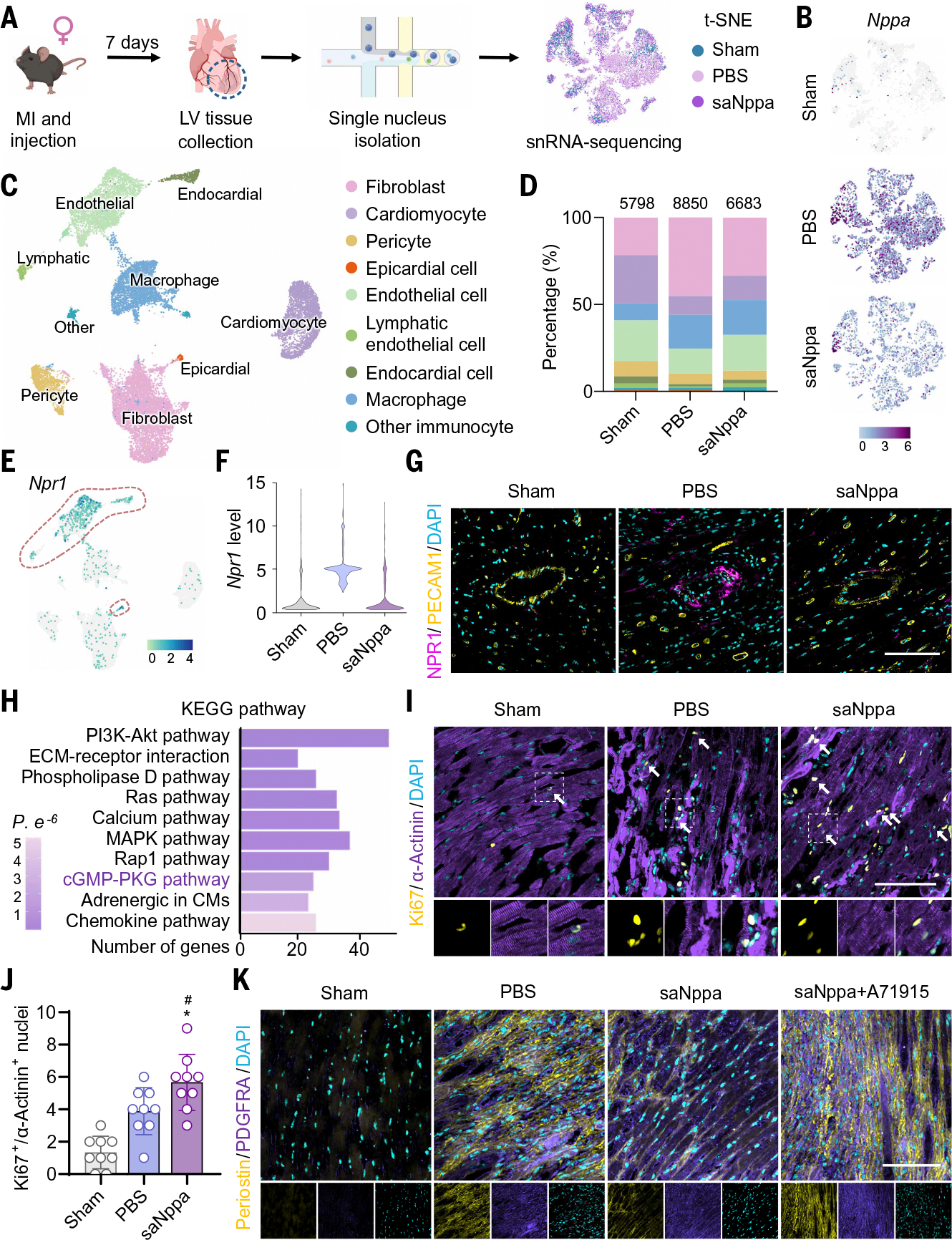
snRNA-seq uncovered mechanism of saNppa therapy. (**A**) Diagram shows the process of sample collection and snRNA-seq using the 10x Genomics platform. [Figure created with BioRender.com] (**B**) Log-normalized *Nppa* expression projected onto t-SNE plots. The color gradient ranges from purple (high expression) to gray (no expression). (**C**) UMAP representation of nine clusters from all three groups. Clusters were annotated according to their characteristic gene markers. (**D**) Fractional composition of all cell populations after clustering. (**E**) Log-normalized *Npr1* expression projected onto UMAP plots. *Npr1* highly expressing clusters are circled with red dashed lines. The color gradient ranges from blue (high expression) to gray (no expression). (**F**) Violin plots showing *Npr1* expression in *Npr1*^+^ cells from each group. (**G**) Confocal micrographs of NPR1 (magenta) and PECAM1 (yellow) in the heart on day 7 after MI and saNppa-LNP injection. Nuclei were counterstained with DAPI (cyan). Scale bar, 100 μm. (**H**) Top 10 relevant KEGG signaling pathways enriched in differentially expressed genes in *Npr1*^+^ cells between the saNppa and PBS groups. (**I**) Confocal images and (**J**) quantification of Ki67^+^ (yellow) and α-actinin^+^ (purple) colocalization in heart tissue sections on day 3 after MI. Nuclei were counterstained with DAPI (cyan). Arrows indicate double positive nuclei. Scale bar, 100 μm. Data are presented as mean ± SD. *n* = 9. Statistical analysis was performed using one-way ANOVA with Tukey’s multiple comparison test. *P < 0.05 versus Sham, ^#^*P* < 0.05 versus PBS. (**K**) Confocal micrographs of periostin (yellow) and platelet-derived growth factor receptor α (PDGFRα) (purple) in the heart on day 7 after MI and saNppa-LNP injection. Nuclei were counterstained with DAPI (cyan). Scale bar, 100 μm.

To elucidate the mechanisms of saNppa-LNP therapy at single-cell resolution, we performed snRNA sequencing on LV tissues collected from female sham-operated mice and MI mice treated with PBS or saNppa-LNP at day 7 after MI (Fig. 3A and Fig. 4A). In total, 21,331 high-quality nuclei were captured and subjected to unsupervised clustering with dimensionality reduction by t-distributed stochastic neighbor embedding (t-SNE) and uniform manifold approximation and projection (UMAP). Analysis of endogenous *Nppa* expression revealed broad induction across multiple cardiac cell types after MI, but *Nppa* levels were markedly reduced after saNppa-LNP injection (Fig. 4B), which is consistent with qPCR validation (Fig. 2H). All nuclei were clustered unsupervised and annotated into nine clusters (Fig. 4C and fig. S10A) according to established marker genes (44, 45) (figs. S10, B and C, and S11). MI caused a loss of CMs, endothelial cells (ECs), and pericytes, while driving expansion of fibroblasts (FBs) and macrophage infiltration. By contrast, saNppa-LNP treatment mitigated CM and EC loss while limiting FB expansion (Fig. 4D), suggesting broad cardioprotective effects.

We next focused on *Npr1*^+^ cells, the principal effector population for ANP signaling. *Npr1* expression largely overlapped with *Pecam1* and *Wt1*, indicating enrichment in endothelial lineage cells (endothelial, endocardial, and lymphatic ECs) and epicardial cells (Fig. 4E and fig. S10B). *Npr1* mRNA levels were elevated in PBS-treated hearts but returned to near normal after saNppa-LNP therapy (Fig. 4F and fig. S12A), a finding confirmed by NPR1/PECAM1 costaining (Fig. 4G) and consistent with negative feedback regulation of the ANP/NPR1 pathway observed above (Fig. 2H and Fig. 4B). In line with increased cGMP levels in LV tissues (Fig. 2J), Kyoto Encyclopedia of Genes and Genomes (KEGG) pathway analysis of *Npr1*^+^ cells showed strong enrichment of cGMP-PKG (PKG, protein kinase G) signaling in saNppa-LNP–treated hearts (Fig. 4H), confirming activation of downstream ANP signaling. To further investigate the modulation of saNppa-LNP on *Npr1*^+^ cells, we performed gene set enrichment analysis of the differentially expressed genes between groups and found that saNppa-LNP treatment was associated with reactivation of regenerative pathways that are suppressed by MI, including Notch, Myc, mTORC, and angiogenesis signaling, alongside attenuation of MI-induced stress-related signatures such as interleukin-6 (IL-6), hypoxia, inflammation, DNA repair, and interferon responses (fig. S12B). These transcriptional changes suggested a potential proregenerative microenvironment after saNppa-LNP treatment. Accordingly, we examined cardiac regenerative capacity after saNppa-LNP treatment using IF staining and found that the numbers of Ki67^+^/α-actinin^+^ and p-H3^+^/α-actinin^+^ CMs were increased (Fig. 4, I and J, and fig. S13A), indicating that saNppa-LNP treatment enhanced CM proliferative capacity. Further gene analysis in *Npr1*^+^ cells confirmed that several well-documented paracrine factors promoting CM proliferation (*Jag1*, *Fgf12*, *Igf2*, *Comp*, *Igf1*, *Vegfa*, *Pdgfb*, *Fgf2*, *Hgf*, and *Pdgfa*) were up-regulated after treatment, whereas a few factors (*Nrg1*, *Kitl*, and *Fgf1*) were down-regulated (fig. S13B). CM proliferation scores also validated up-regulation of proliferation-associated genes in saNppa-LNP treated CMs compared with control groups (fig. S13C). Given the elevated expression of multiple growth factors in *Npr1*^+^ cells, we inferred that saNppa-LNP treatment reprograms the paracrine profile of *Npr1*^+^ cells, thereby transforming the injured microenvironment into a proregenerative, proangiogenic, and anti-inflammatory state that enhances CM regenerative capacity. To better define CM heterogeneity, we identified six CM subclusters (CM1 to CM6) on the basis of marker gene signatures (46, 47) (fig. S13, D and E). According to the gene feature and cell number distribution of CM subclusters, CM2, CM3, and CM6 represented injury-responsive subtypes localized to the infarct border zone (fig. S13, E to G), which is consistent with the high *Nppa* expression observed in border-zone CMs (Fig. 1, C and D). saNppa-LNP treatment reduced the abundance of extracellular matrix (ECM)–secreting CM3 cells, whereas pseudotime trajectories revealed a shift of injured CMs (CM2 and CM3 in the PBS-treated MI group) toward healthy states (CM1 and CM4 in the sham-operated group) after treatment (fig. S13H). Given the pronounced FB changes between PBS and saNppa-LNP groups, we next analyzed FB modulation from saNppa-LNP. Seven FB subclusters (FB1 to FB7) were identified according to the expression of distinct marker gene sets (48–50) (fig. S14, A and B). Resident FBs (FB1/*Negr1*^+^ and FB4/*Gsn*^+^) were present across all groups, whereas FB2 (*Ddr2*^+^), FB3 [periostin-positive (*Postn*^+^)], FB5 (*Mki67*^+^), FB6 (*Wt1*^+^), and FB7 (*Cd36*^+^) expanded after MI (fig. S14, C and D). Pseudotime trajectories suggested that FB3 (*Postn*^+^) cells emerge early after injury and subsequently transition into ECM-secreting FB2 cells, whereas saNppa-LNP treatment markedly reduced the abundance of FB2 and FB3 (fig. S14C). Further cell-cell interaction analysis revealed strong communication between *Npr1*^+^ cells and FB3 (fig. S14E). Analysis of the secreted ligands associated with top 10 signaling pathways in *Npr1*^+^ cells revealed that saNppa-LNP treatment notably down-regulated profibrotic ligands such as *Sema3e*, *Fgf1*, *Bmp5*, *Tgfb2*, *Dll4*, *Col4a1*, and *Col5a1* (fig. S14F). Pseudobulk *z*-score confirmed that saNppa-LNPs suppressed gene expression of ECM components and ECM maturation enzymes, especially profibrosis regulators in *Postn*^+^ FB3 cells (fig. S15). Several dynamic remodeling markers (*Acta2*, *Lgals3*, *Cthrc1*, and *Spp1*) were mildly up-regulated, which is consistent with an early reparative stage in which active remodeling is needed for wound contraction and angiogenesis. Periostin (encoded by *Postn*) immunostaining on day 7 confirmed abundant *Postn*^+^ FBs in infarct border zones after MI, which were markedly reduced by saNppa-LNP treatment. ECM depositions also appeared looser and more organized in treated hearts. Coadministration of the ANP antagonist A71915 reversed these effects, leading to excessive *Postn*^+^ FB accumulation and disorganized ECM (Fig. 4K).

To determine whether saRNA is required for achieving cardioprotective effects, we compared saNppa RNA with conventional nonreplicating *Nppa* mRNA (mNppa) (fig. S16, A and B). A single injection of mNppa-LNP failed to produce significant cardioprotective effects, whereas a single saNppa-LNP injection markedly improved both cardiac function and histological outcomes (fig. S16, C to F). These findings highlighted that self-amplification is essential for achieving therapeutic benefit with a single administration of *Nppa* RNA therapy, underscoring the advantage of saRNA over conventional mRNA.

## saNppa-LNPs provided cardioprotective efficacy across various MI models with comorbidities and delayed treatment

We next asked whether delayed saNppa-LNP administration remained effective under clinically relevant conditions. Unlike immediate treatment, in this model mice received 0.25 mg/kg saNppa-LNPs by IM injection on day 7 after MI. Cardiac function was monitored weekly (fig. S17A). Consistent with above results (Fig. 3B and fig. S8B), body weight loss within the first 3 days after saNppa-LNP injection was still observed (fig. S17B). Before treatment, LVEF had declined to ~25% on day 7 but progressively recovered to ~35 to 40% after a single saNppa-LNP injection. On day 28 after MI, saNppa-LNP treatment considerably improved MI-induced LVAW thinning and LV chamber dilation (fig. S17, C to F). However, strain orbit and GLS analyses indicated no significant recovery of LV strain abnormalities, suggesting that long-term risk of adverse events remained (fig. S17, E and F). Consistently, Masson’s trichrome staining showed that although scar area was larger with delayed saNppa-LNP treatment compared with immediate administration, it was still markedly reduced relative to PBS (*P* = 0.0063) or saFluc (*P* = 0.0014) control, indicating significant antifibrotic effects (fig. S17, G and H). Together, these data indicate that immediate administration of saNppa-LNPs maximizes cardiac preservation and prognosis but that delayed injection still provides meaningful benefits.

To evaluate reproducibility under clinically relevant comorbidities, we tested saNppa-LNPs in three additional MI models. These included an aged MI model using 18-month-old mice (~65 human years) (fig. S18), an atherosclerosis MI model using *Apoe* knockout mice fed with a Western diet for 16 weeks (fig. S19), and a metabolic syndrome MI model induced by high-fat diet plus low-dose streptozotocin to mimic type 2 diabetes (fig. S20). In all three models, a single IM injection of saNppa-LNPs consistently produced reproducible cardioprotective effects, improving LVEF and reducing fibrotic area (figs. S18 to S20). In addition, we also tested therapeutic efficacy in an ischemia/reperfusion (I/R) model that mimics clinical revascularization strategies such as percutaneous coronary intervention or coronary artery bypass grafting. saNppa-LNPs again showed robust cardioprotective effects, with even stronger benefit due to the moderate injury caused by I/R (fig. S21). Because safety is a key concern for saRNA-LNP therapy, we assessed both immune responses and systemic toxicity after injection. Within 24 hours of injection, inflammatory cytokines were measured in muscle tissue and serum. Cytokines such as interferon-γ (IFNγ), granulocyte-macrophage colony-stimulating factor (GM-CSF), tumor necrosis factor–α (TNFα), IL-6, chemokine C-X-C motif ligand 1 (CXCL1), and C-C motif ligand 2 (CCL2) were strongly elevated in muscle tissue, by 10- to 100-fold, whereas IFNγ, IL-6, and CCL2 only rose modestly (five- to 10-fold) in serum (fig. S22). These results suggest that saRNA-LNP injections trigger a transient local inflammatory response and limited systemic cytokine elevation, which is consistent with previous reports for mRNA-LNP administration (51–53). Distinguished from muscle tissue and serum, cytokine levels in LV tissue were largely independent of saRNA-LNP injection and instead strongly associated with MI injury itself. Moreover, treatment with saNppa-LNP partially reduced MI-induced elevations of CXCL2, GM-CSF, and TNFα in LV tissue, suggesting attenuation rather than exacerbation of cardiac inflammation (fig. S22). This profile reinforces the safety advantage of IM injection over direct myocardial delivery. We next examined adaptive immune responses. Serum collected on day 28 revealed no differences in immunoglobulin G (IgG) levels, but on day 3, IgM levels were slightly elevated in the saNppa-LNP group, which likely reflected innate immune stimulation by the saRNA platform and high-level pro-ANP expression that could transiently perturb peripheral tolerance (fig. S23A). Enzyme-linked immunosorbent spot (ELISpot) analysis of splenocytes on day 28 showed no increase in TNFα- or IFNγ-producing T cells compared with controls (fig. S23B), indicating that exogenous native pro-ANP did not elicit adaptive immunity. Lastly, systemic toxicity was evaluated by blood chemistry and histopathological analysis. Neither serum injury markers nor hematoxylin and eosin (H&E) staining of major organs and injection site muscle showed any abnormalities on day 28 after MI (fig. S24, A to F).

## Single IM injection of saNppa-LNPs protected heart function in a swine model of I/R injury

**Fig. 5.**
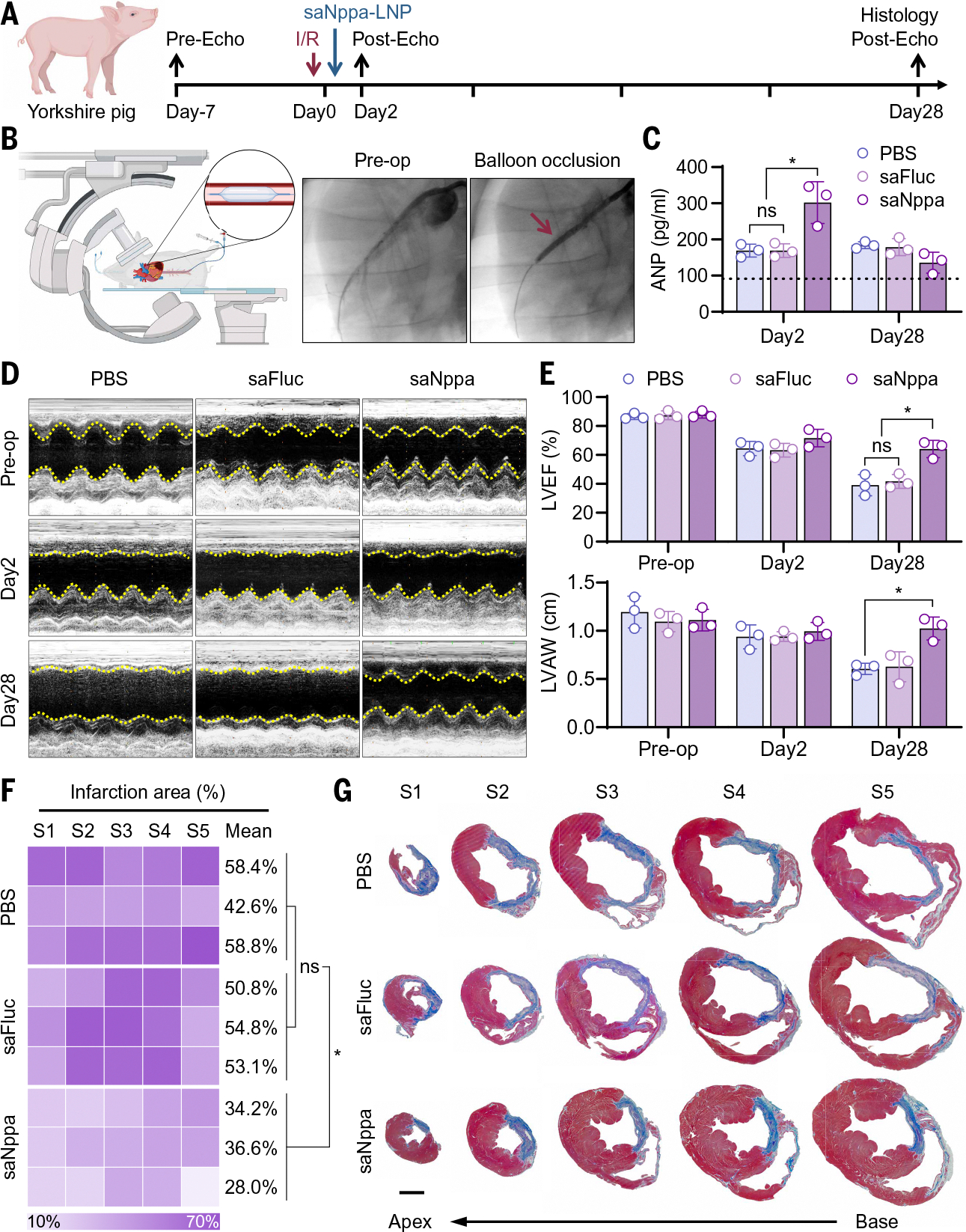
Cardiac protection of saNppa-LNPs in a swine ischemia/reperfusion model. (**A**) Schematic representation of the experimental timeline. A swine I/R model was induced in Yorkshire pigs by 90-min balloon occlusion intervention followed by reperfusion. During recovery, 20 μg/kg of saNppa-LNPs were intramuscularly injected. Cardiac function was evaluated by echocardiogram preoperation and on days 2 and 28 post-MI. Cardiac structure was assessed by histological analysis on day 28. (**B**) Diagram and fluoroscopic images showing establishment of the swine I/R model by balloon occlusion. The balloon location is indicated by a red arrow. [Figure created with BioRender.com] (**C**) ANP levels in pig serum on days 2 and 28. Data are presented as mean ± SD. *n* = 3. Statistical analysis was performed using one-way ANOVA with Tukey’s multiple comparison test. *P < 0.05 between indicated groups. (**D**) Representative echocardiographic images taken preoperation and on days 2 and 28 after MI. (**E**) Measurements of LV ejection fraction (LVEF) and LV anterolateral wall (LVAW) thickness. Data are presented as mean ± SD. *n* = 3. Statistical analysis was performed using one-way ANOVA with Tukey’s multiple comparison test. *P < 0.05 between indicated groups. (**F**) Heatmap showing quantification of infarction area and (**G**) representative images of Masson’s trichrome staining of heart sections from five distinct levels (S1 to S5) of the same heart in each group. Data are presented as mean and individual. *n* = 3. Statistical analysis was performed using one-way ANOVA with Tukey’s multiple comparison test. *P < 0.05 between indicated groups. Scale bar, 10 mm.

To assess the clinical translation potential of saNppa-LNPs, we evaluated their cardioprotective effects in a swine I/R model. The I/R model was established by balloon occlusion of the LAD for 90 min and reperfusion as previously described (54, 55), followed by IM injection of saNppa-LNPs at 20 μg/kg (500 μg per 25 kg pig) (Fig. 5A and fig. S25A). Infarction was confirmed by real-time C-arm x-ray imaging and electrocardiography (Fig. 5B and fig. S25B). Serum ANP levels increased ~1.7-fold after MI, and saNppa-LNP treatment further elevated levels to approximately threefold by day 2, confirming robust exogenous pro-ANP expression after saNppa-LNP injection (Fig. 5C). Cytokine analysis revealed mild increases in transforming growth factor–β, IL-6, and IFNγ, with notable interindividual variability (fig. S25C). Echocardiography demonstrated that a single saNppa-LNP injection markedly rescued LV function, restoring LVEF and normalizing LVAW (Fig. 5, D and E). By day 28 after MI, gross morphology and Masson’s trichrome staining showed reduced fibrosis and prevention of maladaptive remodeling in treated hearts compared with PBS- or saFluc-LNP–injected groups (Fig. 5, F and G, and fig. S25D). Further blood chemical and muscle tissue H&E staining revealed no observable abnormalities on day 28 after injection, supporting long-term systemic safety (fig. S25, E to I). In summary, a single IM injection of saNppa-LNPs provided excellent cardioprotective effects and prevented cardiac fibrosis in a swine I/R model, demonstrating potential promise for clinical translation.

## Discussion

In this study, we developed an intramuscular saRNA-LNP (saNppa-LNP) therapy encoding *Nppa* that enables durable production of cardioprotective pro-ANP. A single injection sustained secretion of pro-ANP for at least 4 weeks, with subsequent cleavage by the cardiac protease corin into active ANP, conferring robust cardioprotection in both murine and porcine MI and I/R models. With snRNA-seq, our mechanistic studies revealed that saNppa-LNP therapy exerted broad cardioprotective effects by reshaping the paracrine activity of *Npr1*^+^ cells, thereby shifting the cardiac microenvironment from profibrotic to proreparative. This favorable milieu supported CM recovery from injury states to healthier phenotypes with increased proliferative capacity, while concurrently suppressing MI-induced expansion of profibrotic *Postn*^+^ and ECM-secreting FBs, resulting in reduced ECM deposition and improved tissue remodeling. saNppa also demonstrated superior efficacy over conventional mRNA at equivalent doses, highlighting the translational potential of this long-acting saRNA platform. Together with validation in comorbidity-associated MI models, delayed treatment efficacy, and longitudinal biosafety, these findings establish saNppa-LNPs as a promising therapeutic approach for heart diseases.

ANP drew our attention at the outset because of its markedly higher induction in neonatal regenerative hearts compared with nonregenerative adult hearts, but the cardioprotective function of natriuretic peptides was already recognized many years ago (31–33, 35, 37). Carperitide, a recombinant human ANP, was previously developed to treat acute MI and heart failure (56). However, clinical trials revealed hypotension and adverse renal effects that limited its use (57). More recently, synthetic agonists of the ANP/NPR1 pathway, including NPR1-activating antibodies, have been explored for cardiovascular therapy (58). We must realize, however, that artificial ANP/NPR1 pathway agonists bypass endogenous feedback networks, raising safety concerns. By contrast, our approach leverages the native pro-ANP protein, allowing precise regulation by the cardiac protease corin and physiological ANP/NPR1 signaling and reducing the risks associated with unrestrained pathway activation.

RNA-LNP technology has been widely used in vaccine and therapeutic approaches for decades (22, 23, 59–64). Compared with vaccine applications of RNA-LNPs, therapeutic protein delivery requires substantially higher expression yields. Accordingly, conventional mRNA-LNP therapeutic applications often use a very high dose; for example, in vivo chimeric antigen receptor T (CAR T) cell engineering typically requires 5 to 10 μg of mRNA per mouse (65, 66), and protein replacement therapy requires even more (around 20 to 30 μg mRNA per mouse) (67). This high dose not only leads to greater cost but also brings unavoidable biosafety concerns. By enabling intracellular amplification, saRNA achieves sustained, high-level protein production at much lower doses. In our study, a single 5 μg saNppa-LNP injection improved cardiac function after MI, underscoring the platform’s efficiency for therapeutic applications. Nonetheless, innate immune responses triggered by saRNA necessitate rigorous biosafety evaluation before clinical translation.

We also observed a distinct biodistribution profile of our nucleotide-modified saRNA-LNPs compared with both conventional modified mRNA-LNPs and unmodified saRNA-LNPs. After IM administration, robust reporter expression was detected predominantly at the injection site (~956.6-fold increase), accompanied by only minimal signal in the draining lymph nodes (~1.9-fold increase). By contrast, prior studies have reported broader tissue expression, including draining lymph nodes, spleen, liver, heart, and other organs, after IM injection of modified mRNA-LNPs (68) or unmodified saRNA-LNPs (69). Moreover, intravenous administration of our saFluc-LNPs resulted in no detectable luciferase signal throughout the mouse as previously reported (70), whereas intravenously delivered mRNA-LNPs typically exhibit prominent hepatic and splenic accumulation with additional signal in lymphoid tissues (71). Together, these findings suggest that nucleotide-modified saRNA-LNPs may follow a distinct in vivo fate compared with mRNA-LNP formulations, potentially reflecting differences in protein corona formation, cellular uptake, or intracellular processing. Further studies will be required to delineate the mechanisms governing these spatiotemporal distribution patterns.

Another critical barrier to clinical translation of RNA-based cardiac therapy has been the delivery route. Previous mRNA therapy for heart diseases often relied on direct cardiac injection (4, 7). However, both the injection procedure and the drug itself may further stress an already injured heart. By contrast, our intramuscular approach leverages the heart-targeted prodrug nature of pro-ANP, enabling safe and simple systemic delivery. This strategy not only avoids direct myocardial manipulation but also opens the possibility of developing saNppa-LNPs as an emergency intervention that could be administered in prehospital settings. Our use of FDA-approved LNP formulations and demonstration of efficacy in large-animal I/R models further strengthen the clinical translatability of saNppa-LNPs. Our work demonstrates the promise of the application of saRNA in regenerative medicine, offering an effective and clinically applicable approach for the potential treatment of heart disease.

## Materials and methods

### RNA design and synthesis

The backbone plasmid of the saRNA template used in this study was modified from T7-VEE-IRES-Puro, a gift from Steven Dowdy (RRID: Addgene 58970; https://n2t.net:443/addgene:58970) (72). The IRES and puromycin coding sequences were deleted when mouse *Nppa* (NM_008725.3), pig *NPPA* (NM_214260.2), GFP, or firefly luciferase coding sequences were inserted into the backbone. The resulting plasmids were named T7-VEE-mouseNppa, T7-VEE-pigNppa, T7-VEE-GFP, and T7-VEE-Fluc, respectively. Then, these template plasmids were linearized with MluI digestion for IVT. For the synthesis of conventional mRNA (mouse *Nppa*, GFP and Fluc mRNAs), the templates were PCR-amplified fragments containing a T7 promoter. All IVT templates were purified either by phenol-chloroform extraction or using the Monarch PCR & DNA Cleanup Kit (T1030, New England Biolabs, NEB).

Both conventional mRNA and saRNA used in this study were synthesized with 5-methyl-CTP and pseudouridine-UTP modifications. IVT was performed using HiScribe T7 Quick High Yield RNA Synthesis Kit (E2050, NEB). 5-Methyl-CTP (B7967, ApexBio Technology) and pseudouridine-UTP (B7972, ApexBio Technology) were added to the IVT reaction mixture and the final molar ratio of modified nucleotides to standard nucleotides was 1:2. Subsequently, the synthesized RNAs were capped with Faustovirus Capping Enzyme (M20081, NEB) and tailed with *E. coli* Poly(A) Polymerase (M0276, NEB). After each step, the reaction products were purified using Monarch RNA Cleanup Kit (T2050, NEB). All synthesized RNAs were analyzed by agarose gel electrophoresis to check the integrality. The final verified RNAs were resuspended in RNA Storage Solution (AM7000, Invitrogen) at 1 mg/ml and aliquoted for storage at −80°C.

### LNP formulation and characterization

LNP formulations were prepared using a self-assembly process as previously described (73). In this study, we tested three ionizable cationic lipids to deliver saRNA, including Dlin-MC3-DMA (HY-112251, MedChemExpress, MCE), ALC-0315 (HY-138170, MCE), and SM-102 (HY-135541, MCE). DSPC (850365P, Avanti), Cholesterol (700100P, Avanti), DMG-PEG 2000 (880151P, Avanti) and ALC-0159 (HY-138300, MCE) were also used in the formulations. The detailed formulations were listed in table S1. In general, lipids were dissolved in ethanol and RNA were resuspended in 25 mM sodium acetate (pH 5.2). The nitrogen:phosphate (N:P) ratio was set to 6 for each formulation. The lipid mixture was mixed into the RNA solution at a volume ratio of 1:3 (ethanol: aqueous) manually followed by the dialysis against PBS (pH 7.2) at 4°C for 4 to 6 hours. The prepared LNPs should be used within 3 days of 4°C storage.

Particle size and ζ potential of LNPs were determined by NanoSight NS300 (Malvern Panalytical) and Malvern Particle Size Analyzer (Malvern Panalytical). RNA loading efficiency was calculated by using modified RiboGreen RNA kit (R32700, Invitrogen). Briefly, naked or LNP encapsulated saRNAs were broken down by ribonuclease A (R1253, Thermo Scientific) and then residual RNAs were extracted with TRIzol reagent (15596026, Invitrogen) following the manufacturer’s instructions. The undegraded RNAs were quantified by RiboGreen RNA kit as shown as loading percentage. Morphology of LNPs was observed with Talos Arctica Cryo-EM (Thermo Scientific). To assess the transfection efficiency of LNPs, 1 × 10^5^ HEK 293 cells were seeded into a 12-well plate and cultured to 50% confluence. Then, 100-ng GFP saRNA encapsulated by different LNP formulations were added to the culture medium for 48 hours. Transfection efficiency was analyzed and calculated with Flow Cytometry. Expression duration time was also detected in HEK 293 cells. After 100-ng GFP saRNA or mRNA, loaded LNPs were added into 12-well plate cultured HEK 293 cells, and images were captured at the indicated time point with ECHO Revolve microscope.

### Enzyme-linked immunosorbent assay (ELISA)

To measure pro-ANP levels in Fig. 2G, 5 μg of mouse saNppa-LNPs were IM injected into healthy mice. On day 3 after injection, serum was collected for subsequent analysis. Serum from healthy untreated mice served as the control. To measure pro-ANP and cGMP levels in Fig. 2, I and J, 5 μg of mouse saNppa-LNPs were intramuscularly injected into MI mice. PBS was injected as the buffer control. On day 3 after MI and injection, mice were euthanized and perfused with 20 ml of ice-cold PBS, after which LV tissues were collected. Then LV tissues were minced to small pieces and homogenized in PBS containing protease and phosphatase inhibitor cocktail (Thermo Scientific, 78441) using a microtissue grinder. The resulting suspension underwent two freeze-thaw cycles to further lyse the cells. After that, the homogenates were centrifuged, and the supernatants were collected for subsequent measurements. For mouse serum pro-ANP assay in Fig. 3C, 0.25 mg/kg saNppa-LNPs were intramuscularly injected into MI mice; saFluc-LNPs and PBS served as controls. At the indicated time points, blood was collected to prepare serum samples. All samples were aliquoted and stored at −80°C until assay performance. Serum samples were appropriately diluted, and 10 μg of total protein from tissue homogenates was used for each assay. Mouse pro-ANP ELISA kits (MODL00971, MOES00492, AssayGenie) and cGMP ELISA kits (4360, Cell Signaling Technology) were used according to the manufacturers’ instructions.

For porcine serum ANP assay in Fig. 5C, 20 μg/kg of pig saNppa-LNPs were intramuscularly injected into pigs. At the indicated time points, blood was collected to prepare serum samples. All samples were aliquoted and stored at −80°C until assay performance. Serum samples were diluted as required and analyzed using the Porcine Natriuretic peptides A (NPPA) ELISA Kit (PREB0083, AssayGenie).

### Real-time qPCR

Total RNA was extracted from the border zone of LV ischemia tissues in each group using TRIzol reagent (15596026, Invitrogen) following the manufacturer’s instructions. Then, cDNA was synthesized using iScript cDNA Synthesis Kit (Bio-Rad, 1708891). SsoAdvanced Universal SYBR Green Supermix (Bio-Rad, 1725271)-based real-time qPCR analysis was performed with a Bio-Rad CFX Opus 96 Real-Time PCR System. Relative mRNA expression changes were calculated using the 2^−ΔΔCt^ method and normalized to *Actb*. Each independent experiment was performed in duplicate. Primers used here were listed following: *Actb*: forward primer sequence, 5′-3′, CCCCTGAACCCTAAGGCCA; reverse primer sequence, 5′-3′, CGGAGTCCATCACAATGCCT; *Nppa*: forward primer sequence, 5′-3′, GAGTGGACTAGGCTGCAACA; reverse primer sequence, 5′-3′, TCAAGCAGAATCGACTGCCT.

### Western blotting

Cell and tissue samples were lysed in RIPA buffer with protease and phosphatase inhibitor cocktail (Thermo Scientific, 78441) and then quantified by using BCA assay (Thermo Scientific, 23225). Then, 20 to 40 μg of total protein were separated on Mini-PROTEAN TGX Stain-Free Precast Gels (Bio-Rad) and transferred onto 0.22-μm polyvinylidene fluoride (PVDF) membranes (Bio-Rad). Subsequently, the membrane was blocked in 5% non-fat milk and incubated with the following primary antibodies: anti-β-Actin (MA5–33078, Invitrogen), anti-α-Tubulin (ab7291, Abcam), anti-GFP (ab290, Abcam), anti-ANP (ab225844, Abcam), and anti-Corin (711422, Invitrogen). After incubation with horseradish peroxidase-conjugated secondary antibodies, the enhanced chemiluminescence kit (Bio-Rad, 1705061) was used to image the membrane with a ChemiDoc Imaging System (Bio-Rad). Original images of all blots are shown in fig. S26.

### Multimodal imaging

Both in vivo and ex vivo imaging experiments were performed as previously described (74–76). For the investigation of injection routes, 5 μg saFluc-LNPs were administered via IV, IM, or SC injection respectively. For the expression duration study, 5 μg of saFluc-LNPs or mFluc-LNPs (~50 μl) were intramuscularly injected into hindlimbs of the C57BL/6J mice. At the indicated time points, mice were intraperitoneally injected with D-luciferin (150 mg/kg; Revvity) and then imaged within Spectral Instruments Imaging’s AMI HTX system. For the ex vivo Fluc imaging, the organs were collected then washed in chilled PBS following capturing the images immediately. Fluc signals were measured and analyzed using Aura Imaging Software (Spectral Instruments Imaging). To visualize the saNppa-LNPs in vivo, saNppa-LNPs were labeled with DiR dye (1 μM, HY-D1048, MCE) during fabrication and dialyzed to remove extra free dye. DiR-labeled LNPs were intramuscularly injected into hindlimbs, and in vivo living imaging and ex vivo organ imaging of DiR signals were performed with Spectral Instruments Imaging’s AMI HTX system or IVIS Lumina Imaging System (excitation, 745 nm; emission, 810 nm) and measured by the radiant efficiency of regions of interest by using Aura Imaging Software or Living Image software.

### Mouse model

In this study, C57BL/6J mice (male and female, 6 to 10 weeks), and aged C57BL/6J mice (male, 18 months), were purchased from the Charles River Laboratories. The *Apoe* knockout mice (B6.129P2-*Apoe*^tm1Unc^/J, male, 6 weeks) were purchased from the Jackson Laboratory. All animal studies complied with the ethical regulations of Columbia University, under the approval of IACUC Protocol AC-AABX4650. Pain relief medicines were administered according to Protocol AC-AABX4650.

### Mouse experiments

#### Neonatal mouse experiments:

Neonatal MI model was established on day 1 after birth as previously reported (77, 78). The neonatal mice were anesthetized by hypothermia, and the heart was then exposed for LAD artery ligation (8–0). The sham-operated mice were subjected to the same procedure without ligating of the LAD.

To knock down *Nppa* expression in neonatal mouse hearts, a mixture of three AAV9 vectors encoding shRNAs targeting mouse *Nppa* (AAV9-Nppa-shRNA cocktail) was prepared. Following the successful establishment and survival of the MI model, each mouse received a single retro-orbital injection of 1E11 genome copies of the AAV9-shRNA cocktail on the same day. Control mice were injected with an equivalent dose of AAV9 carrying a non-targeting shRNA (AAV9-NC). One week after injection, LV border-zone tissues were harvested for qPCR and IF staining to evaluate *Nppa* knockdown efficiency. At 28 days after MI, echocardiography was performed, and hearts were collected for subsequent histological analysis.

#### Adult mouse experiments:

The MI model was established as previously described (79, 80). Briefly, mice were prepared by removing hair and administering eye ointment. Then mice were intubated under anesthesia, followed by thoracotomy to expose the heart. The LAD was ligated by 6–0 suture. After confirming LV tissue turned pale, the chest wall and incision were closed by 4–0 suture.

Aged MI model was established using 18-month-old male C57BL/6J mice with LAD ligation.

Atherosclerosis MI model was established using *Apoe* knockout mice, as previously described (81). Male mice were fed with a Western diet (21% fat, 0.2% cholesterol) for 16 weeks to induce atherosclerosis. Following dietary induction, MI was surgically induced by LAD ligation.

Metabolic syndrome MI model was established using male C57BL/6J male C57BL/6J mice (82, 83). Mice were fed with a high-fat diet for 12 weeks and subsequently received three consecutive daily intraperitoneal injections of streptozotocin (STZ, 50 mg/kg; dissolved in freshly prepared 0.1 M citrate buffer, pH 4.5) to induce type 2 diabetes. Fasting blood glucose concentrations were monitored, and only mice with glucose concentrations >200 mg/dl were included in subsequent procedures. MI was then induced by LAD ligation.

The I/R model was established as previously reported (54). Briefly, male C57BL/6J mice were prepared by hair removal and administration of ophthalmic ointment. Then mice were intubated under anesthesia, followed by thoracotomy to expose the heart. The LAD was occluded with a 6–0 suture for 45 min and then released to allow reperfusion. Chest wall and incision were closed with 4–0 sutures. The sham-operated mice underwent the same procedure without LAD ligation.

To inhibit ANP signaling in vivo, the ANP receptor antagonist A71915 was administered using a continuous delivery system. Briefly, A71915 was delivered via subcutaneously implanted osmotic minipumps (Alzet model 1004), which provided sustained release of the antagonist at a rate equivalent to 60 μg/kg/day. Pumps were prepared and primed according to the manufacturer’s instructions before implantation. At the designated experimental time points, mice were euthanized and heart tissues were collected for subsequent histological analyses.

#### Injection routes, time, and dose:

The optimal injection route of saRNA-LNP was determined using saFluc reporter RNA. In brief, 5 μg of saFluc-LNPs were administered via IV, IM, or SC injection respectively. On the first 3 days, Fluc signals were monitored by living image with D-luciferin (150 mg/kg; Revvity) using Spectral Instruments Imaging’s AMI HTX system.

For dose response analysis of saNppa-LNP treatment, 0.05, 0.25, or 0.5 mg/kg of saNppa-LNPs were intramuscularly injected into the hindlimb muscles on the same day after MI. PBS was intramuscularly injected as buffer control. Cardiac function and structure were assessed on day 28 after MI and injection.

For all therapeutic effect experiments, 0.25 mg/kg of saNppa-LNPs were intramuscularly injected into the hindlimb muscles in a total volume of approximately 50 μl after MI, and 0.25 mg/kg of saFluc-LNPs were intramuscularly injected as vehicle control. The same volume of PBS was intramuscularly injected as the buffer control. Care was taken to avoid puncturing blood vessels or leakage of LNPs into the circulation. The sham-operated mice underwent the same procedure without LAD ligation. Body weight was recorded, and blood samples were collected at the indicated time points. Cardiac function and structure were assessed on day 28 after MI and injection.

To compare the cardioprotective efficiency of saNppa-LNP versus conventional mNppa-LNP, 0.25 mg/kg of saNppa-LNPs or mNppa-LNPs were intramuscularly injected after MI. PBS was injected as the buffer control. The sham-operated mice underwent the same procedure without LAD ligation. Cardiac function and structure were assessed on day 28 after MI and injection.

In the delayed treatment experiment, female C57BL/6J mice underwent MI surgery and on day 7 after MI, 0.25 mg/kg of saNppa-LNPs were intramuscularly injected into the hindlimb muscles. Cardiac function was monitored weekly by echocardiography, and heart tissues were collected on day 28 after MI for further histological analysis.

### Swine model

A swine I/R model was established in Yorkshire pigs (female, 23 to 25 kg) by balloon occlusion intervention as described previously (54, 55). In brief, after the pig was anesthetized, a femoral catheter was placed percutaneously using aseptic technique. Cardiac catheterization was performed with C-arm. Fluoroscopy images were captured to clarify the infarct position of the artery; before, during and after occlusion. Myocardial infarction was induced by inflation of an angioplasty balloon (TREK(R) OTW 1.5 to 3 mm, Abbott Vascular, Santa Clara, CA or similar system) in the mid LAD distal to the second diagonal branch for 90 min. During the recovery, 20 μg/kg of saNppa-LNPs (500 μg RNA per 25 kg pig) were intramuscularly injected into the muscle tissues in a total volume of around 2 to 3 ml. Same dose of saFluc-LNPs (20 μg/kg) were intramuscularly injected as vehicle control. Same volume of PBS was also intramuscularly injected as buffer control. Echocardiography and blood sample collection were performed before operation and on day 2 and 28 after MI. Heart tissues were collected on day 28 after MI for further histological analysis. All animal studies complied with the ethical regulations of Columbia University, under the approval of IACUC Protocol AC-AABY0651.

### Echocardiogram

Cardiac function was measured by transthoracic echocardiograph using Visulasonics VEVO 3100 High Frequency Ultrasound Imaging System (MX550D probe for mice) or Phillips EPIQ 7 Ultrasound System (S5–1 probe for pigs). Briefly, at indicated time points, the mouse was anesthetized by 2% isoflurane to capture the B-mode images and M-mode images of both short-axis and long-axis. Then, LVAW thickness, LV diameter at the end diastole and systole, and LVEF were measured by Vevo LAB software. The strain orbit and GLS were further analyzed by Vevo Strain software. In pigs, transthoracic echocardiography was performed to acquire short-axis B-mode and M-mode images, and LVEF and LVAW thickness were quantified using ImageJ software. All measurements were acquired from three continuous cardiac cycles.

### Histopathology and immunostaining

At indicated time points, mice were euthanized and perfused with chilled PBS. Tissue samples were collected and fixed in 10% neutral buffered formalin for 24 hours. Following dehydration with gradient ethanol, hyalinization with xylene, tissues were embedded in paraffin and cut into 5-μm paraffin sections. For cryosection, tissues were dehydrated with a 30% sucrose solution, embedded in Optimal Cutting Temperature (OCT) compound, and sectioned to a thickness of 5 μm. H&E staining and Masson’s trichrome staining were performed with paraffin sections according to a standard protocol.

Cryosections were used to do immunofluorescence staining. In brief, cryosections were blocked with 10% normal goat serum and incubated with primary antibodies against ANP (1:200; ab225844, Abcam), α-Actinin (1:200; ab9465, Abcam), Ki67 (1:200, ab15580, Abcam), p-Histone H3 (1:200, ab5176, Abcam), Periostin (1:200, ab14041, Abcam) or CD68 (1:200; 137001, BioLegend) at 4°C overnight, followed by incubation with secondary antibodies labeled with Alexa Fluor 488, Alexa Fluor 594, or Alexa Fluor 647 (Abcam) at room temperature for 2 hours. DAPI was used to visualize nuclei. Imaging was captured by using a Nikon Ti2 inverted microscope with an AXR resonant spectral scanning confocal unit or an ECHO Revolve microscope.

### snRNA-seq

Female C57BL/6J mice were used here for snRNA-seq. After the MI model was established, 0.25 mg/kg of saNppa-LNPs were intramuscularly injected into the hindlimbs. Sham-operated mice and MI mice injected with PBS served as controls. On day 7 after MI and treatment, mice (3 mice per group) were euthanized and perfused with 20 ml of ice-cold PBS. The LV tissues were collected and quickly frozen in liquid nitrogen as previously reported (84). Then the frozen tissue samples were submitted to Singulomics Corporation for single nuclei isolation and further librarying (10x Genomics) and sequencing. Single-nucleus expression data were processed using the Cell Ranger Single Cell Software Suite (version 7.0.1) to perform quality control, sample demultiplexing, barcode processing, and single-nuclei 3′ gene counting by the vender. Data integration was performed by Seurat (version 5.3.0). Unsupervised clustering was performed in Seurat with a resolution of 0.4 using 30 dimensions following the Satija Laboratory Tutorial (https://satijalab.org/seurat/articles/essential_commands). Quality control was performed, and nuclei were filtered based on mitochondrial content (<10%) and genes per nucleus (>200 and <3000). After filtering, 21,331 nuclei were used for further analysis. The gene-nucleus-barcode matrix of the samples was normalized by the number of unique molecular identifiers per nucleus. In brief, differential expression of genes was tested using the Find All Markers function with the statistical test wilcox in the Seurat package for focused analyses. Genes with adjusted *P* values <0.05 were considered as differentially expressed genes. Pseudotime analysis was performed with the tool Slingshot (version 2.14.0). Slingshot infers developmental lineages by linking clusters on a low-dimensional embedding (via a minimum-spanning tree) and fitting simultaneous principal curves along those links, thereby ordering cells in pseudotime to describe transitions between states. Intercellular communication analysis was conducted using the CellChat R package (version 2.2.0). CellChat was used to infer and analyze the cell-to-cell communication networks within the dataset. To do so, we computed cell-cell communication probabilities and identified key signaling pathways among *Npr1*^+^ cells and FB subclusters. Communication probabilities were calculated using the compute Commun Prob and compute Commun Prob Pathway functions, while pathway-level communications were visualized using circle plots. The interaction strength among clusters was quantified, and differential expression of ligands was aggregated across all three groups. For the FB3 subcluster, we generated pseudobulk profiles by summing raw counts per sample within FB3, followed by library-size normalization and log-transform (log_2_CPM). We computed gene-wise *z*-scores across samples (center and scale by gene) for heatmap visualization. Differential expressions within FB3 were tested with Seurat’s FindMarkers (Wilcoxon), reporting log_2_ fold-change and multiple-testing adjusted *P* values.

### Inflammatory cytokine array

Male C57BL/6J mice were used for cytokine array. After MI, 0.25 mg/kg of saNppa-LNPs or saFluc-LNPs were intramuscularly injected into hindlimbs. The same volume of PBS was intramuscularly injected as buffer control. Sham-operated mice and sham-operated mice intramuscularly injected with 0.25 mg/kg of saNppa-LNPs also served as additional controls here. Sera, LV, and muscle tissues were collected within 24 hours (~16 to 18 hours) after saRNA-LNP injection. LV and muscle tissues were homogenized in RIPA buffer containing protease and phosphatase inhibitor cocktail (Thermo Scientific, 78441). Prepared serum and tissue homogenate samples were submitted to Eve Technologies Corporation (Calgary, Canada) for analysis using the mouse high sensitivity 18-PLEX discovery assay (MDHSTC18).

Porcine cytokine assays were performed using sera collected on day 2 after MI and IM injection of 20 μg/kg of saRNA-LNPs. Collected serum samples were analyzed with the Porcine Cytokine Array GS5 (GSP-CYT-5–1, RayBiotech). Data acquisition and analysis was performed by RayBiotech.

### Serum IgM and IgG detection

Serum samples were collected from mice on day 3 or day 28 after MI and 0.25 mg/kg of saRNA-LNP injection. To measure anti-pro-ANP IgM, 96-well ELISA plates were coated overnight at 4°C with recombinant mouse pro-ANP protein (5 μg/ml) in carbonate bicarbonate buffer, followed by blocking with 1% BSA. Serum collected on day 3 after MI was diluted 1:10 in PBS and incubated on the coated plates, and bound IgM was detected using HRP-conjugated anti-mouse IgM secondary antibody. Sera from sham-operated mice were used as control. To measure anti-pro-ANP IgG, serum collected on day 28 after MI was diluted 1:10 in PBS and incubated on plates coated under the same conditions with recombinant mouse pro-ANP (5 μg/ml), and bound IgG was detected using an HRP-conjugated anti-mouse IgG secondary antibody. Optical density at 450 nm (OD450) was measured to quantify antibody levels.

### ELISpot measurement

T cell recall responses were evaluated using a mouse IFN-γ/TNF-α Double-Color FluoroSpot kit (ImmunoSpot) according to the manufacturer’s instructions and as previously described (64). Splenocytes were isolated from each group on day 28 after MI and IM injection of 0.25 mg/kg of saRNA-LNPs. Cells were stimulated with recombinant pro-ANP protein (5 μg/ml). Unstimulated wells served as negative control, and the wells treated with Cell Activation Cocktail (423301, BioLegend) served as positive control. Spots corresponding to IFN-γ and TNF-α secreting cells were developed and quantified using an ImmunoSpot analyzer by ImmunoSpot (Cleveland, OH).

### Chemistry panel analysis

Blood samples from mice and pigs were collected on day 28 after MI and saRNA-LNP injection to isolate the serum. All serum samples were submitted to the diagnostic laboratory of the Institute of Comparative Medicine, Columbia University, for serum chemistry analysis.

### Statistical analysis

All studies were evaluated in at least three independent experiments for each condition to ensure reproducibility. Data are expressed as scatter plots with the mean ± SD or individual values. Significant differences between different groups were determined using two-tailed unpaired Student’s *t*-tests for two-group comparisons and one- or two-way analysis of variance (ANOVA) with post hoc Tukey’s test for multiple group comparisons. Statistical analyses were performed using GraphPad Prism software (GraphPad Software, San Diego, CA) or R. Differences were considered significant at *P* < 0.05.

## Supplementary Material

Supplementary file

science.org/doi/10.1126/science.adu9394

Figs. S1 to S26; Table S1; Reproducibility Checklist

## Data Availability

All data are available in the main manuscript or the supplementary materials. All sequencing raw data are available on GEO with accession no. GSE313956. Requests for materials should be addressed to K.C.
