# Supplementary material for: Single intramuscular injection of self-amplifying RNA of Nppa to treat myocardial infarction
Source: Science. Author manuscript; Available in PMC 2026 Apr 28. (PMC13124201; doi:10.1126/science.adu9394)
Supplement: Supplementary file [file NIHMS2156683-supplement-Supplementary_file.pdf]

Science
AAAS

Supplementary Materials for

Single intramuscular injection of self-amplifying RNA of *Nppa* to treat myocardial infarction

Kaiyue Zhang *et al.*

Corresponding author: Ke Cheng, ke.cheng@columbia.edu

Science **391**, eadu9394 (2026)
DOI: 10.1126/science.adu9394

The PDF file includes:

Figs. S1 to S26
Table S1

Other Supplementary Material for this manuscript includes the following:

Reproducibility Checklist

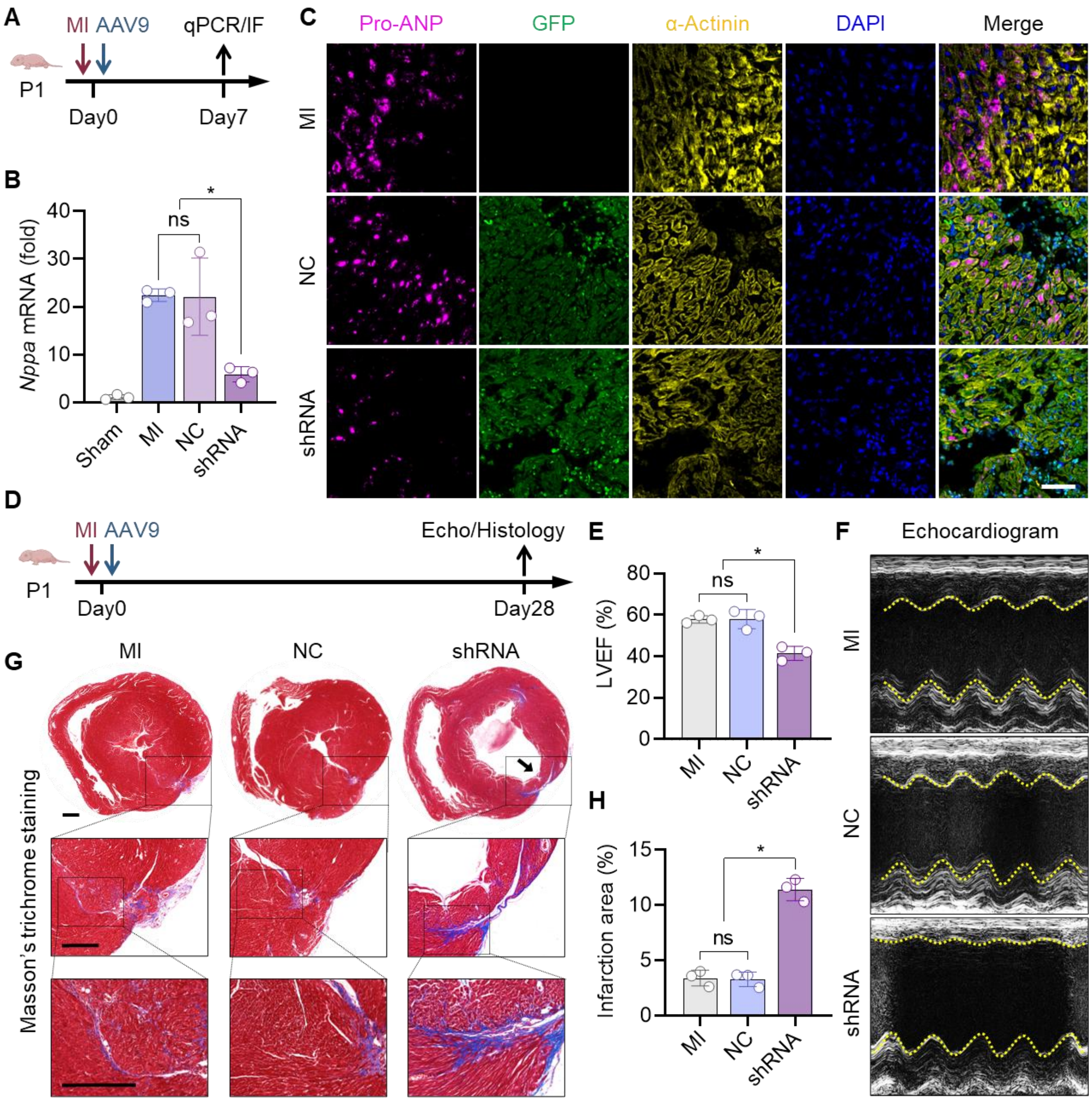

Fig. S1. Nppa is required in neonatal cardiac repair after MI.

(**A**) Schematic illustrating AAV9-shRNA mediated knockdown of *Nppa* and validation in neonatal MI hearts. (**B**) *Nppa* mRNA levels in the border zone of neonatal hearts on day 7 after MI and AAV9-shRNA injection. All data were normalized to samples collected from sham-operated adult mice. Data are expressed as mean ± SD. n = 3. Statistical analysis was performed using one-way ANOVA with Tukey's multiple comparison test. **P* < 0.05 between the indicated groups. ns, not significant. (**C**) Immunofluorescence staining of pro-ANP (magenta) in neonatal MI heart (α-actinin, yellow, cardiomyocytes) with or without AAV9-shRNA injection on day 7 after MI. GFP (green) expressed by AAV9 served as a reporter. Nuclei were counterstained with DAPI (blue). Scale bar, 50 μm. (**D**) Schematic illustrating AAV9-shRNA mediated knockdown of *Nppa* in neonatal hearts subjected to MI and subsequent cardiac regeneration assessments on day 28. (**E**) LVEF of neonatal hearts on day 28 after MI and AAV9 injection. Data are expressed

as mean ± SD. n = 3. Statistical analysis was performed using one-way ANOVA with Tukey's multiple comparison test. *$P < 0.05$ between the indicated groups. ns, not significant. (**F**) Echocardiography images of neonatal hearts on day 28 after MI and AAV9 injection. (**G**) Masson's trichrome staining images of neonatal hearts on day 28 after MI and AAV9 injection. Arrow indicates infarction area with fibrosis. Scale bars, 500 µm. (**H**) Quantification of infarction area from Masson's trichrome staining images. Data are expressed as mean ± SD. n = 3. Statistical analysis was performed using one-way ANOVA with Tukey's multiple comparison test. *$P < 0.05$ between the indicated groups. ns, not significant.

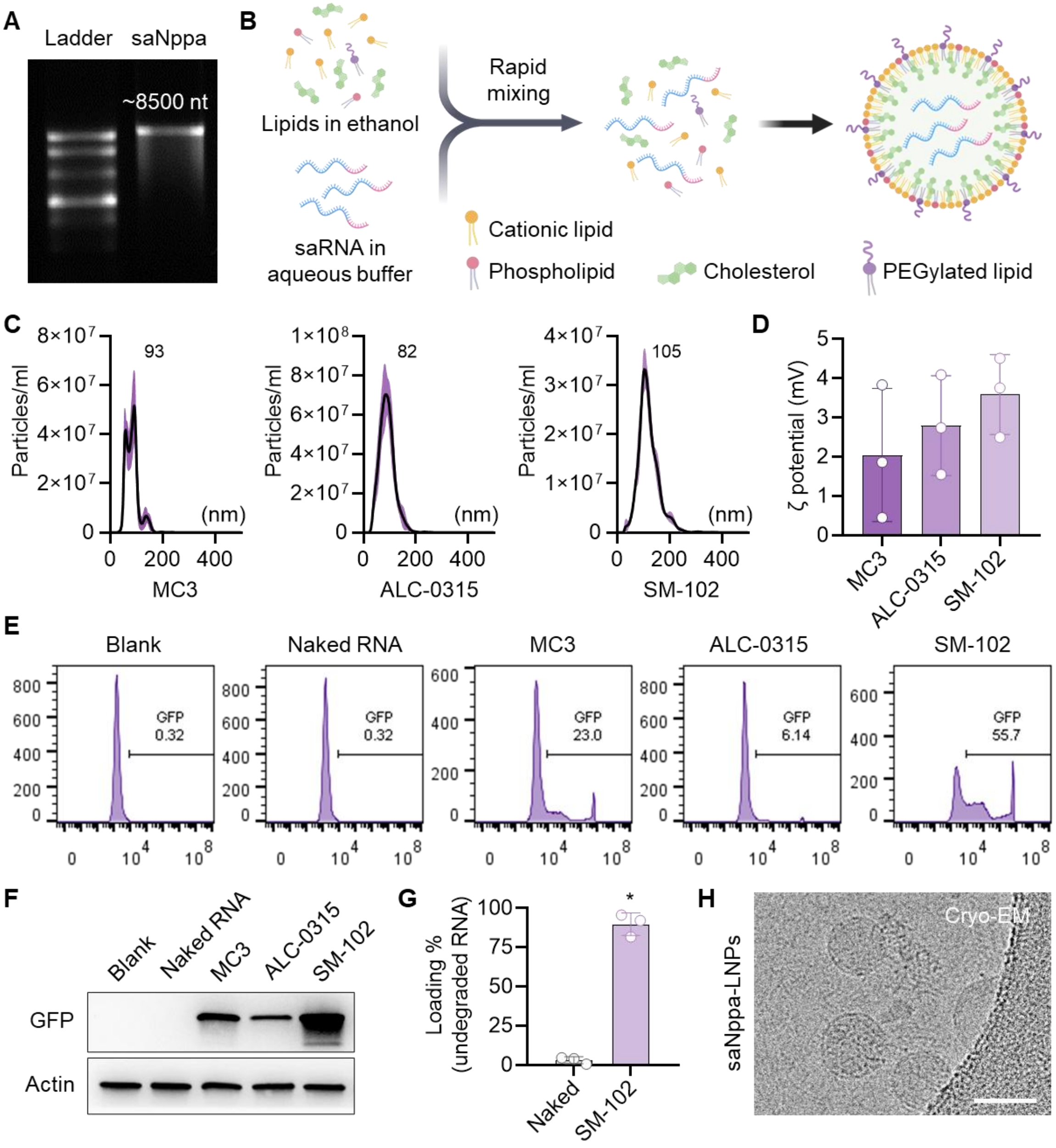

Fig. S2. Formulation and characterization of saRNA-LNPs.

(**A**) Agarose gel electrophoresis image of synthesized saNppa RNA. (**B**) Schematic illustrating the preparation of saRNA-LNPs using a self-assembly process. (**C**) Size distributions and particle numbers of saNppa-LNPs with 3 different formulations. (**D**) ζ potentials of saNppa-LNPs with 3 different formulations. Data are presented as mean ± SD. n = 3. (**E**) Transfection efficiency of saGFP-LNPs with 3 different formulations analyzed by flow cytometry. Naked saGFP RNA served as control. (**F**) GFP production yields of 3 different formulated saGFP-LNPs analyzed by western blotting. (**G**) Loading efficiency of saNppa RNA encapsulated in SM102-formulated LNPs. Data are presented as mean ± SD. n = 3. Statistical analysis was performed using a two-tailed unpaired Student's *t*-test. $^*P < 0.05$ versus naked saNppa RNA. (**H**) Cryo-EM image of SM102-formulated saNppa-LNPs. Scale bar, 100 nm.

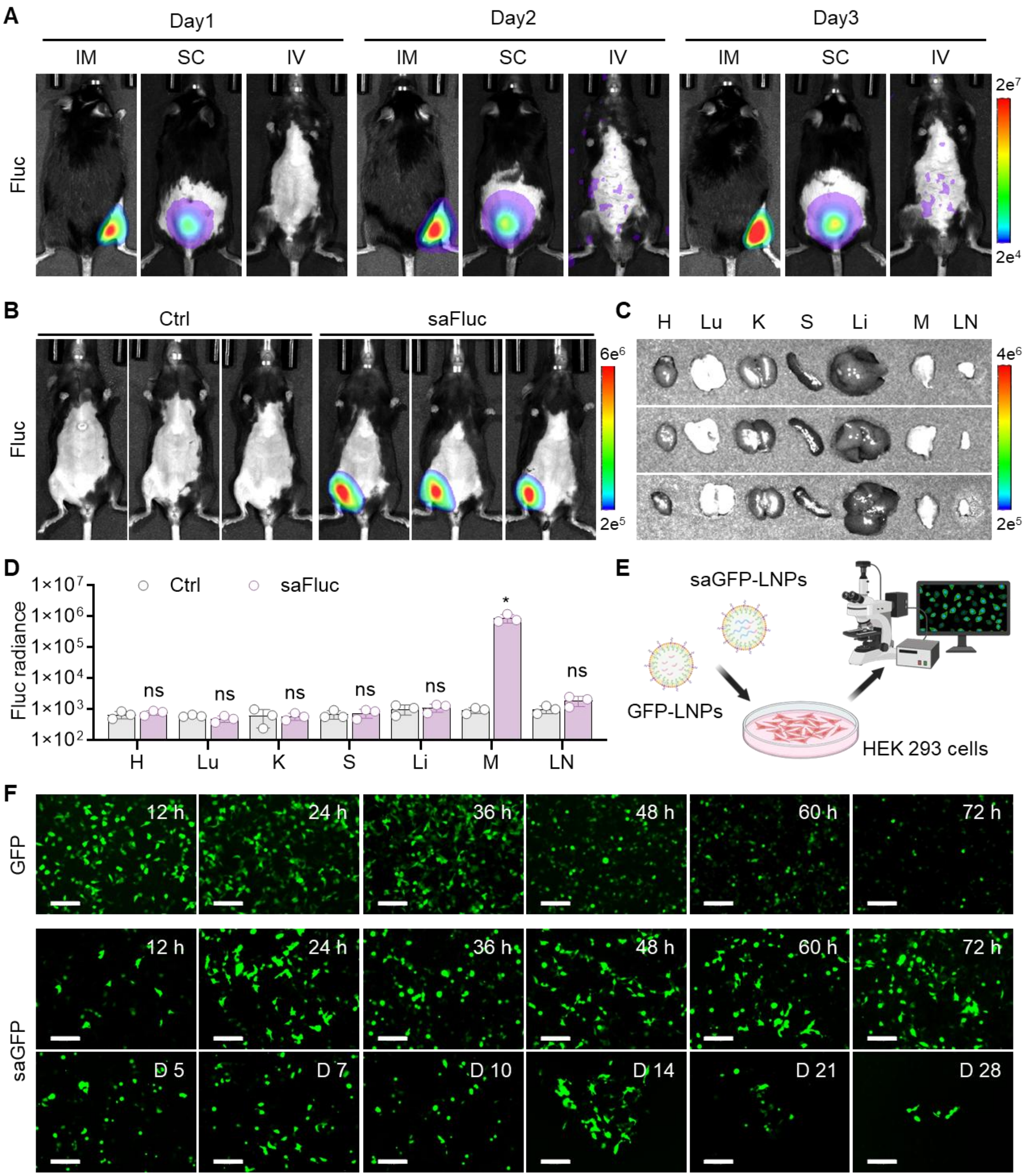

Fig. S3. Injection routes, biodistribution and protein production duration of saRNA-LNPs. (**A**) Representative bioluminescence images of mice after a single IM, SC, and IV injection of 5 µg saFluc-LNPs on day 1, 2, and 3 post-injections. The radiance of Fluc signal is expressed as photons/s. (**B**) Bioluminescence imaging of mice after IM injection of 5 µg saFluc-LNPs or equal volume PBS. (**C**) Ex vivo bioluminescence imaging of main organs collected from PBS injected mice on day 3. H, heart; Lu, lung; K, kidney; S, spleen; Li, liver; M, muscle; LN, lymph node. (**D**) Quantification of Fluc signals from the indicated organs on day 3 after IM injection of PBS or saFluc-LNP. H, heart; Lu, lung; K, kidney; S, spleen; Li, liver; M, muscle; LN, lymph node. The average radiance of the Fluc signal is expressed as photons/s/cm^2/steradian. Data are

presented as mean ± SD. n = 3. Significance was determined by two-way ANOVA with Tukey's multiple comparison test. **P* < 0.05 versus Ctrl. ns, not significant versus Ctrl. (**E**) Diagram of in vitro protein expression duration of saGFP-LNPs examined in HEK 293 cells. GFP mRNA loaded LNPs served as control. (**F**) Duration of GFP expression in HEK 293 cells after transfection with 100 ng GFP mRNA or saGFP RNA. Scale bars, 100 μm.

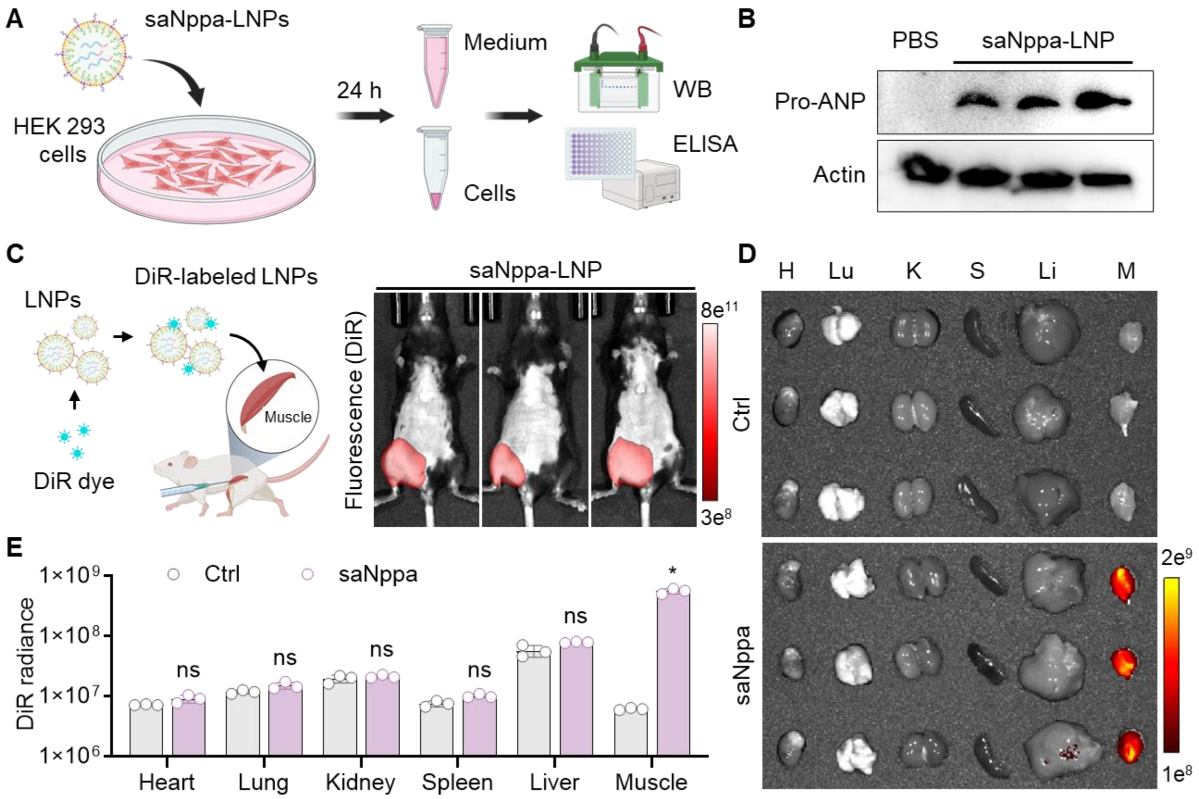

Fig. S4. Pro-ANP expression and biodistribution of saNppa-LNPs.

(**A**) Experiment design for pro-ANP expression and secretion in HEK 293 cells after transfection with 100 ng of saNppa RNA loaded in SM102-LNPs. (**B**) Pro-ANP expression in HEK 293 cell lysates 24 hours after saNppa-LNP transfection in three independent biological replicates. (**C**) Diagram and fluorescence imaging of living mice after IM injection of DiR-labeled saNppa-LNPs. (**D**) Organ distribution of DiR-labeled saNppa-LNPs determined by DiR radiant efficiency on day 3 after IM injection. H, heart; Lu, lung; K, kidney; S, spleen; Li, liver; M, muscle. (**E**) Quantitative analysis of DiR radiant efficiency in the indicated organs on day 3 after injection. The radiant efficiency of DiR was expressed as [photons/s/cm^2/steradian]/[μW/cm^2]. n = 3. Significance was determined by two-way ANOVA with Tukey's multiple comparison test. *$P < 0.05$ versus Ctrl. ns, not significant versus Ctrl.

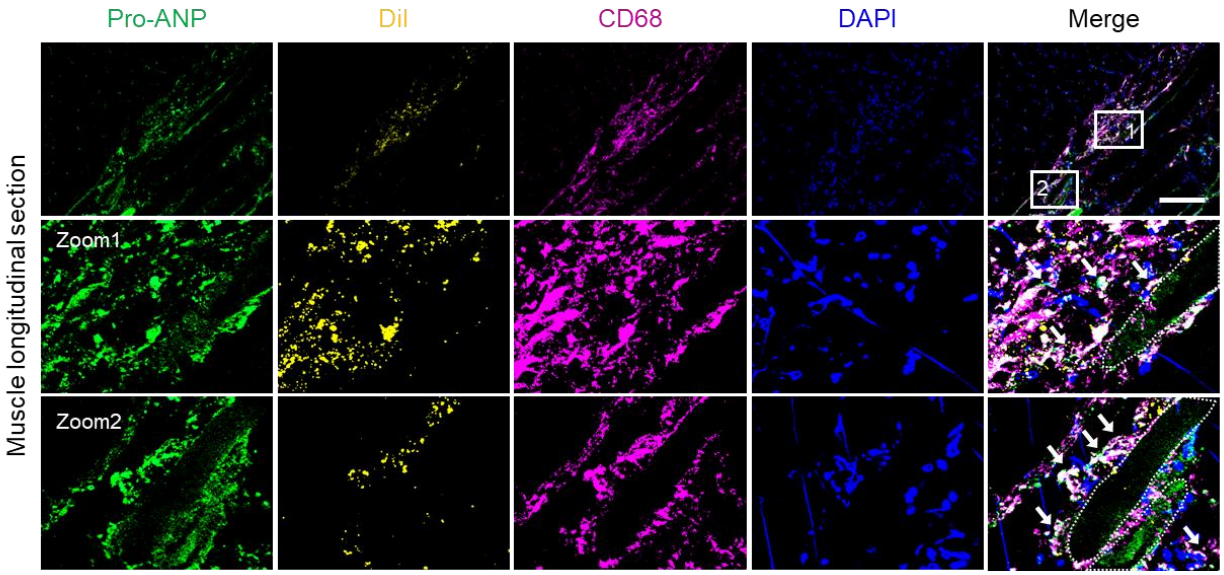

Fig. S5. Pro-ANP expression and cell distribution on day 3 after IM injection.

Representative images and corresponding zoomed-in views of pro-ANP (green), LNPs (DiI, yellow), and CD68 (magenta) in muscle longitudinal section on day 3 after saNppa-LNP injection. Nuclei were counterstained with DAPI (blue). Scale bars, 200 µm. Dashed lines outline pro-ANP expressing skeletal muscle fibers, and arrows indicate macrophages expressing pro-ANP.

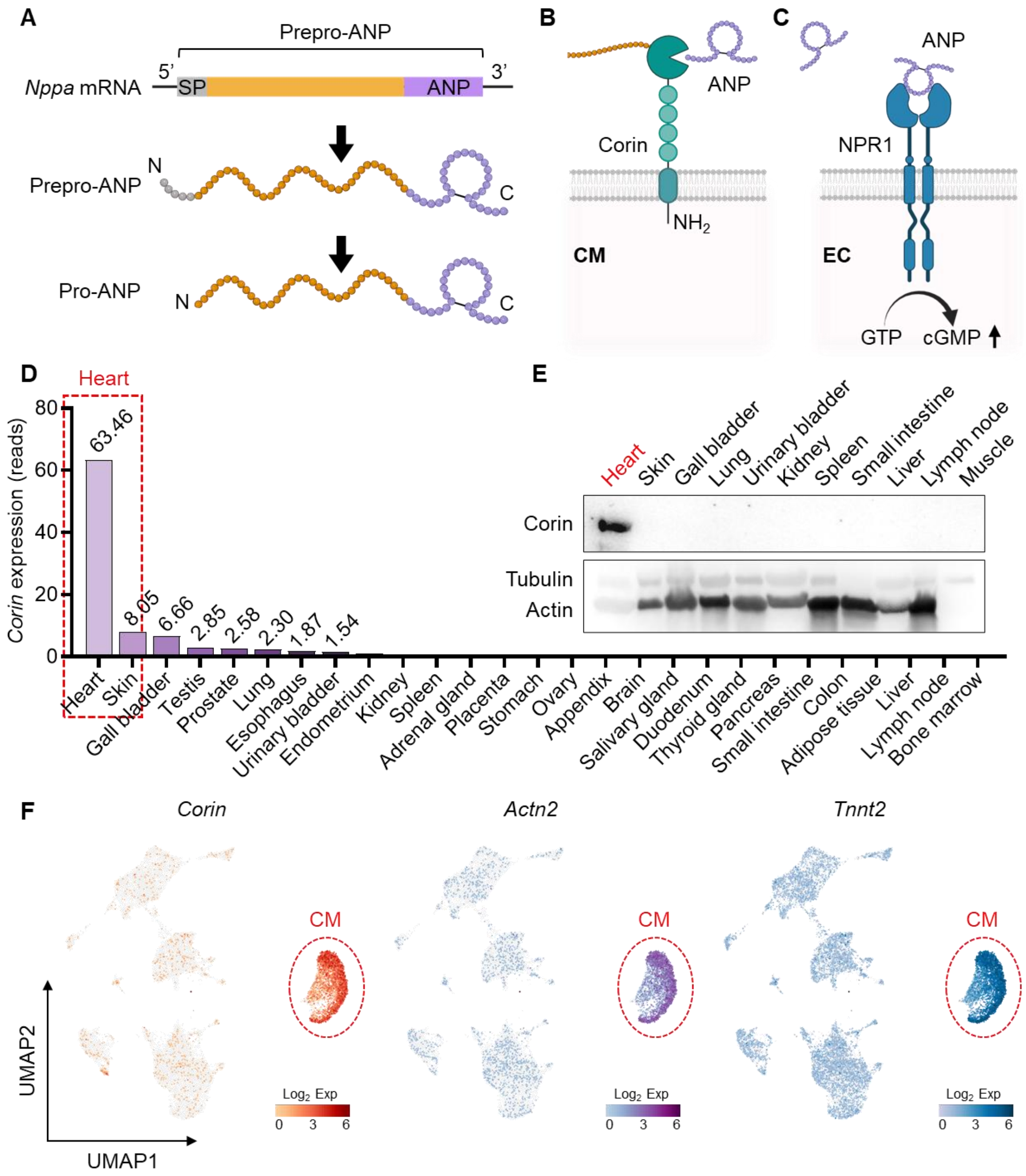

Fig. S6. Corin-mediated pro-ANP processing in the heart.
(**A**) Schematic diagrams of pro-ANP processing. *Nppa* mRNA encodes prepro-ANP, which undergoes removal of its N-terminal signal peptide during intracellular vesicle trafficking to form pro-ANP. (**B**) Pro-ANP is secreted and cleaved by corin, which is a type II transmembrane serine protease anchored on the cardiomyocyte (CM) surface, thereby generating biologically active ANP. (**C**) ANP and its receptor natriuretic peptide receptor 1 (NPR1). The primary receptor of ANP is NPR1, a transmembrane guanylyl cyclase predominantly expressed on endothelial cells (ECs). Upon ANP binding, NPR1 catalyzes the synthesis of cGMP, a key second messenger that regulates cardiac development and regeneration. GTP, guanosine

triphosphate. (**D**) *Corin* mRNA levels across 20 human tissues. The original data were obtained from NCBI database (BioProject: PRJEB4337, Publication: PMID 24309898). (**E**) Western blot analysis of corin expression in mouse tissues. (**F**) *Corin* expression is enriched in CMs (markers: *Actn2* and *Tnnt2*) shown on a UMAP plot. Cells are projected in UMAP space and colored according to the relative expression of each gene. The color gradient ranges from red, blue, and magenta (*Corin*, *Actn2*, and *Tnnt2*; high expression) to gray (no expression).

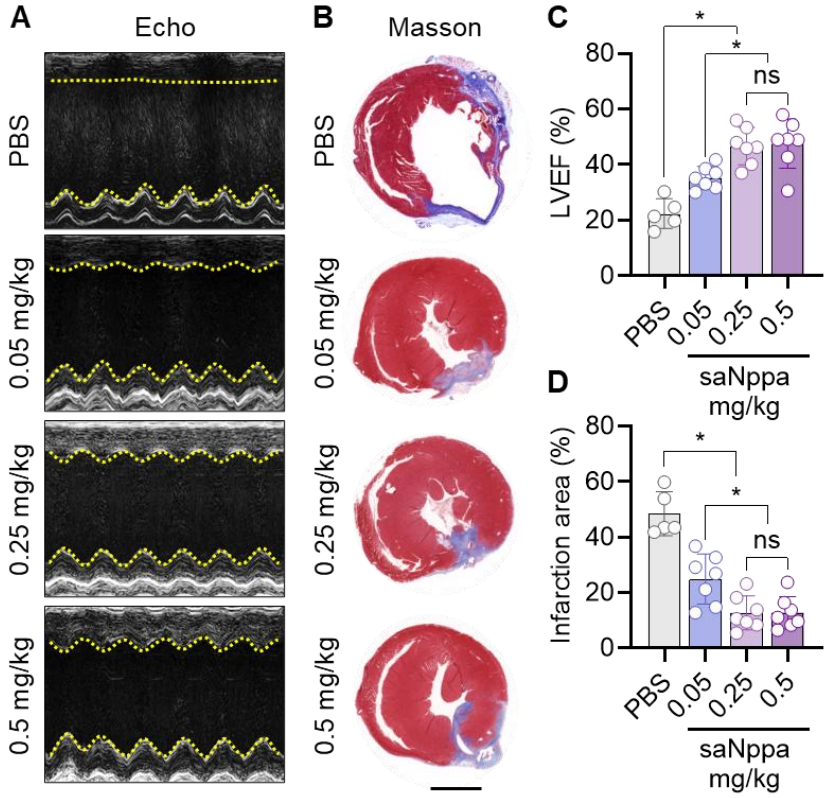

Fig. S7. Dose response of saNppa-LNP therapy.

(**A**) Representative echocardiography images on day 28 after MI and saNppa-LNP injection. (**B**) Representative Masson's trichrome staining images of heart cross sections. Scale bar, 2 mm. (**C**) Quantification of LV ejection fraction (LVEF). Data are presented as mean ± SD. n = 5 or 7. Statistical analysis was performed using one-way ANOVA with Tukey's multiple comparison test. *$P < 0.05$ between indicated groups. (**D**) Quantification of infarction area. Data are presented as mean ± SD. n = 5 or 7. Statistical analysis was performed using one-way ANOVA with Tukey's multiple comparison test. *$P < 0.05$ between indicated groups. ns, not significant.

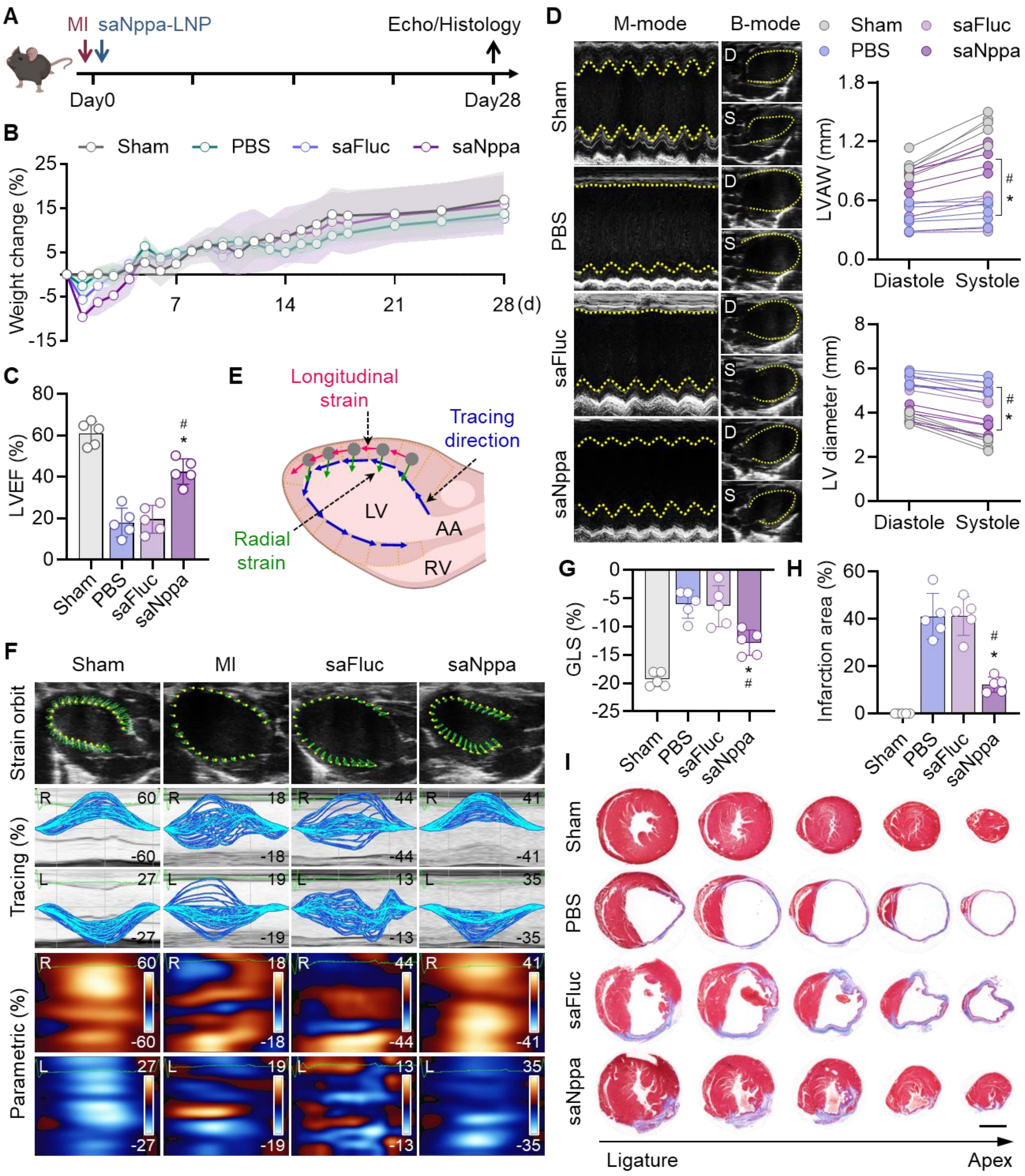

Fig. S8. Cardiac protective effects of saNppa-LNPs in male mice.

(**A**) Schematic representation of the experimental timeline. (**B**) Body weight changes in male mice after MI and treatment. Data are presented as mean ± SD. n = 5. (**C**) Quantification of LV ejection fraction (LVEF). Data are presented as mean ± SD. n = **5**. Statistical analysis was performed using one-way ANOVA with Tukey's multiple comparison test. $*P < 0.05$ versus PBS, $^{\#}P < 0.05$ versus saFluc. (**D**) Representative echocardiography images and measurements of LV anterolateral wall thickness and LV diameter. Data are presented as individual values. n = 5. Statistical analysis was performed using two-way ANOVA with Tukey's multiple comparison test. $*P < 0.05$ versus PBS, $^{\#}P < 0.05$ versus saFluc. (**E**) Schematic illustration of

speckle-tracking-based myocardial strain analysis, showing longitudinal (magenta) and radial (green) deformation of the left ventricle, with endocardial border tracing (blue) used to quantify directional myocardial strain. (**F**) Representative strain orbits (top), strain tracings (middle) and parametric distributions (bottom) of the LV endocardium along the radial and longitudinal axis. Strain tracings (*y* axis) for each speckle are displayed along the traced contour over time (*x* axis). For the radial strain parametric distributions, motion at each speckle is displayed as blue (moving away from the center of the heart) or red (moving towards the center) over time (*x* axis). For the longitudinal strain parametric distributions, motion at each speckle is displayed as blue (moving away from the apex) or red (moving towards the apex). (**G**) Measurements of LV endocardium global longitudinal strain. Data are presented as mean ± SD. n = 5. Statistical analysis was performed using one-way ANOVA with Tukey's multiple comparison test. $^{*}P < 0.05$ versus PBS, $^{\#}P < 0.05$ versus saFluc. (**H**) Quantification of infarction area cross groups. Data are presented as mean ± SD. n = 5. Statistical analysis was performed using one-way ANOVA with Tukey's multiple comparison test. $^{*}P < 0.05$ versus PBS, $^{\#}P < 0.05$ versus saFluc. (**I**) Masson's trichrome staining images of sequential heart cross sections from the ligature site to the apex. Scale bar, 2 mm.

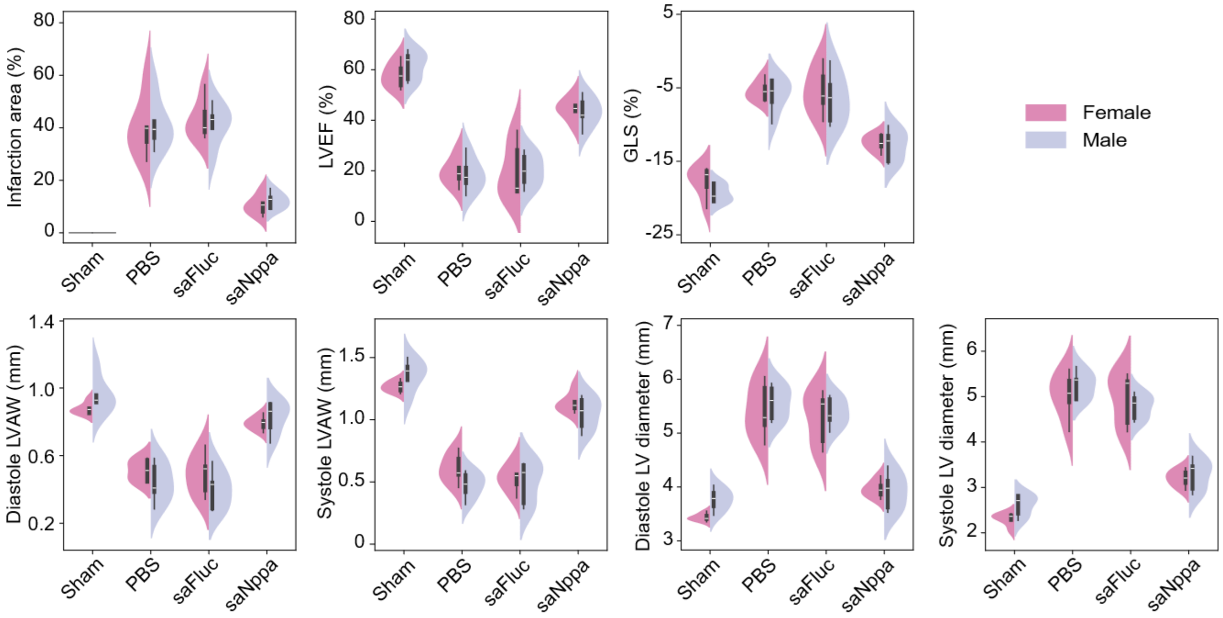

Fig. S9. Combined cardiac functional outcomes of saNppa-LNP therapy in male and female MI mice.

Violin plots summarizing pooled echocardiographic and histological parameters from both female and male MI mouse models treated with PBS, saFluc-LNPs, or saNppa-LNPs. Sham-operated mice served as controls. Parameters include infarction area, LVEF, GLS, LVAW thickness and LV diameter. Data from female (pink) and male (blue) mice were integrated to illustrate overall therapeutic efficacy.

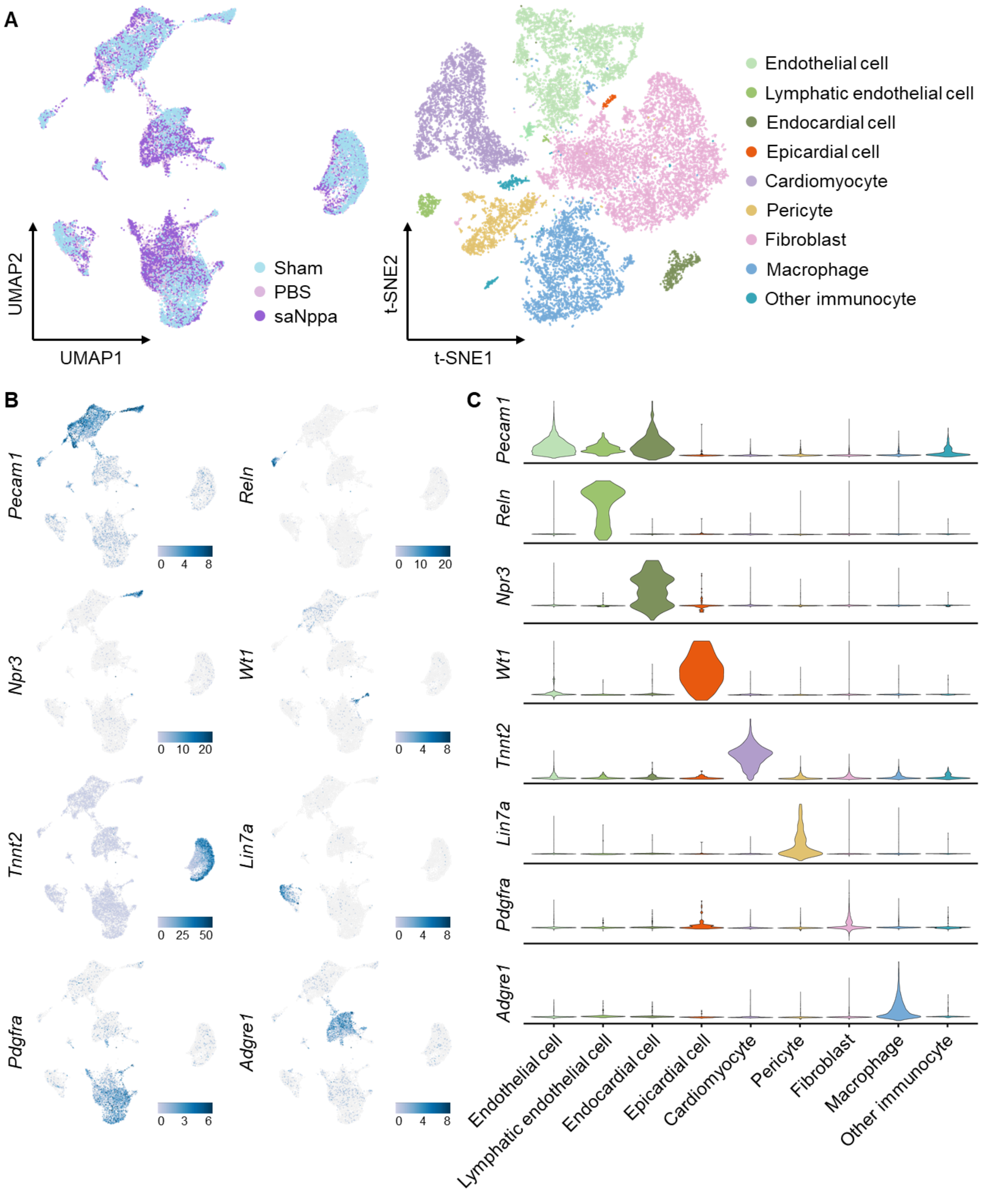

Fig. S10. Cellular composition of the mouse heart after saNppa-LNP treatment.
(**A**) UMAP and t-SNE clustering of 21,331 nuclei after quality control and data filtering, and batch correction using Canonical Correlation Analysis (CCA) integration in Seurat. (**B**) UMAP visualization of representative feature gene expression within each cluster. The color gradient ranges from blue (high expression) to gray (no expression). (**C**) Violin plots generated from the integrated dataset displaying characteristic marker genes of each identified cell cluster.

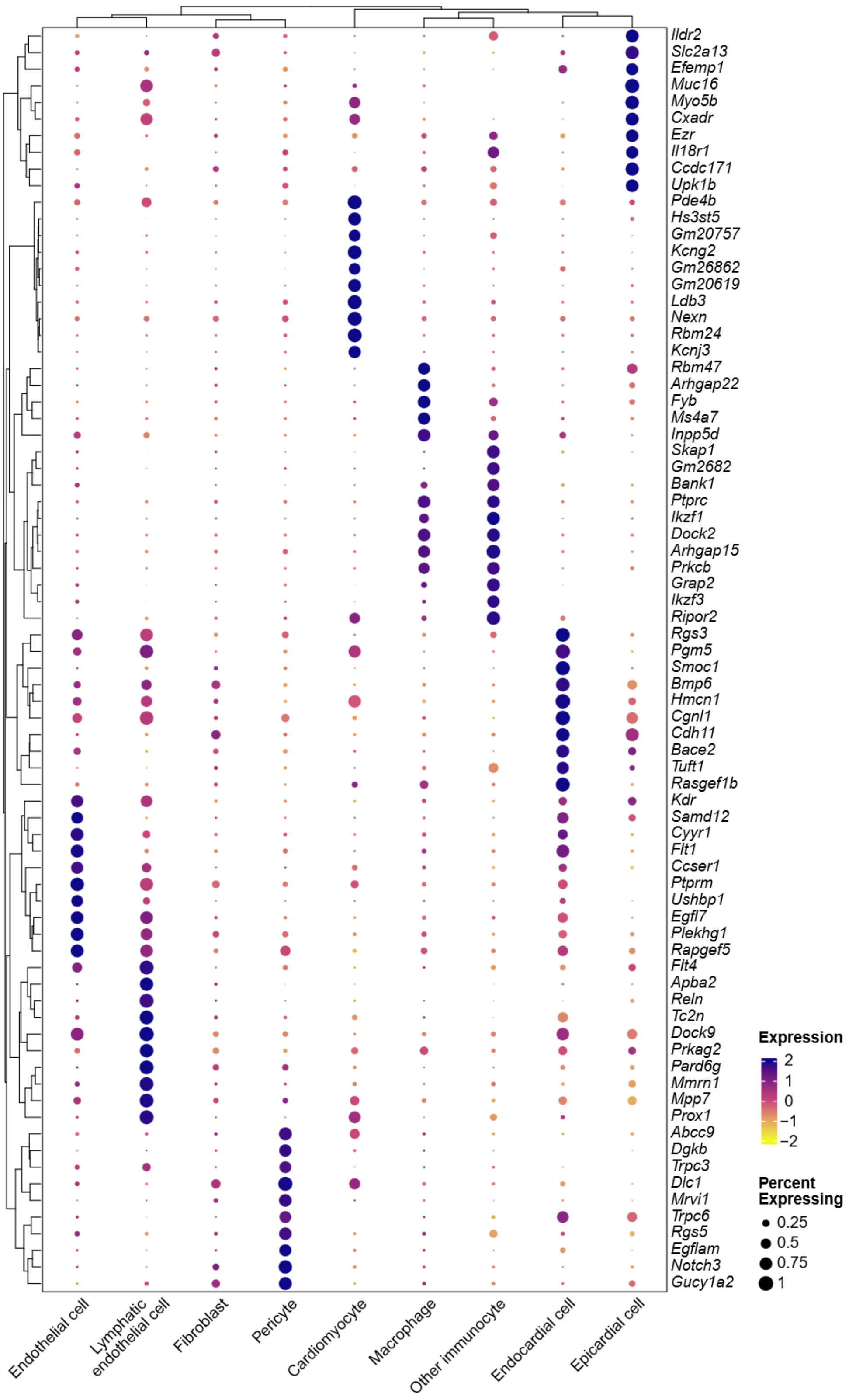

Fig. S11. Gene signatures of each identified cell population.

Top 10 marker genes enriched in each cluster are displayed.

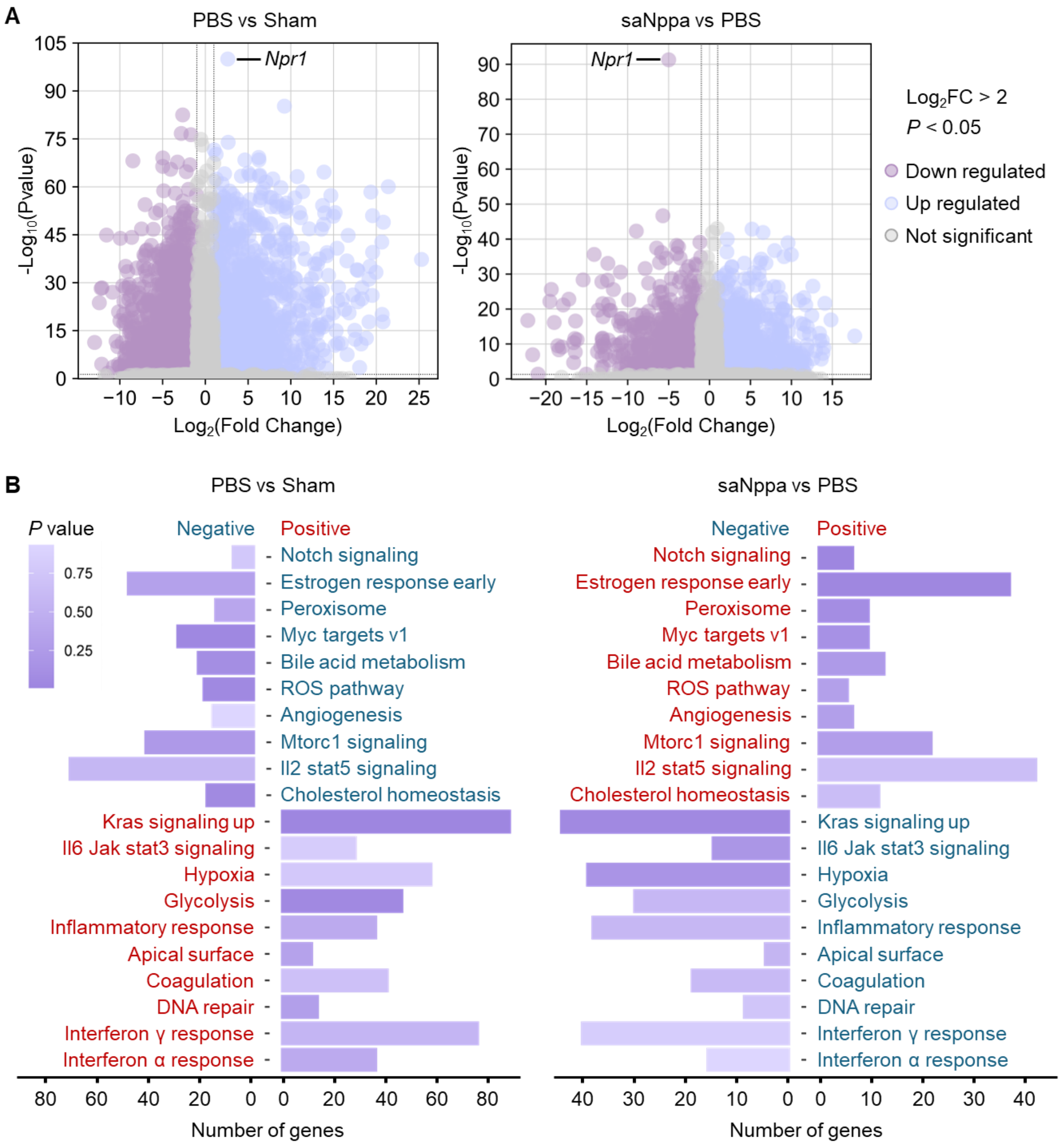

Fig. S12. Differential gene expression analysis of *Npr1*⁺ cells between each group.

(**A**) Volcano plots showing fold changes and *P* values for genes that are up- or down-regulated in comparisons between: MI mice treated with PBS versus sham-operated mice, and MI mice treated with saNppa-LNP versus MI mice treated with PBS. (**B**) Gene Set Enrichment Analysis (GSEA) of Hallmark pathways showing enriched gene sets associated with *Npr1*$^+$ cells from the sham-operated mice and MI mice with PBS or saNppa-LNP treatment. *P* values were calculated by the GSEA software (v4.3, Broad Institute).

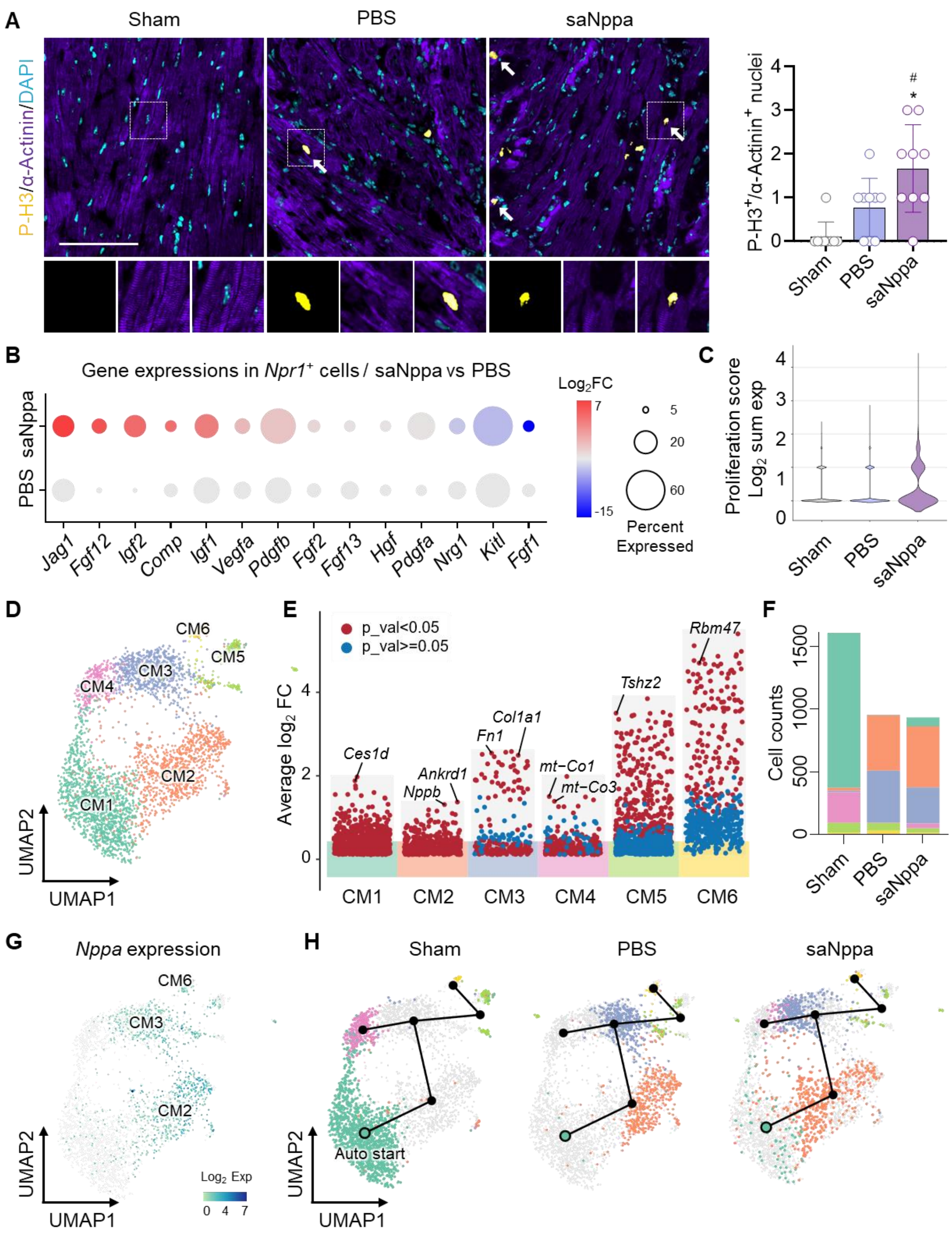

Fig. S13. Proliferative activity and sub-clustering of cardiomyocytes.

(**A**) Confocal images and quantification of p-H3+ (yellow) and α-actinin+ (purple) colocalization in heart tissue sections on day 3 after MI and treatment. Nuclei were counterstained with DAPI (cyan). Arrows indicate double positive stained nuclei. Scale bar, 100 μm. Data are presented as

mean ± SD. n = 9. Statistical analysis was performed using one-way ANOVA with Tukey's multiple comparison test. $^{*}P < 0.05$ versus Sham, $^{\#}P < 0.05$ versus PBS. (**B**) Selected genes (adjusted P value < 0.05) encoding proliferation related paracrine factors in *Npr1*$^{+}$ cells between saNppa and PBS groups. The color bar indicates $\log_2$ fold change of gene expression. Dot sizes represent the percentage of cells expressing genes. (**C**) Violin plots revealing proliferation score of CMs from all groups. (**D**) UMAP visualization of 6 CM subclusters. (**E**) Feature plots of marker genes for each CM subcluster. (**F**) Cell population distributions of CM subclusters delineated into 3 groups and 6 subclusters. (**G**) UMAP visualization of *Nppa* expression in CM subclusters. (**H**) Slingshot plots showing pseudotime trajectories of CM subclusters.

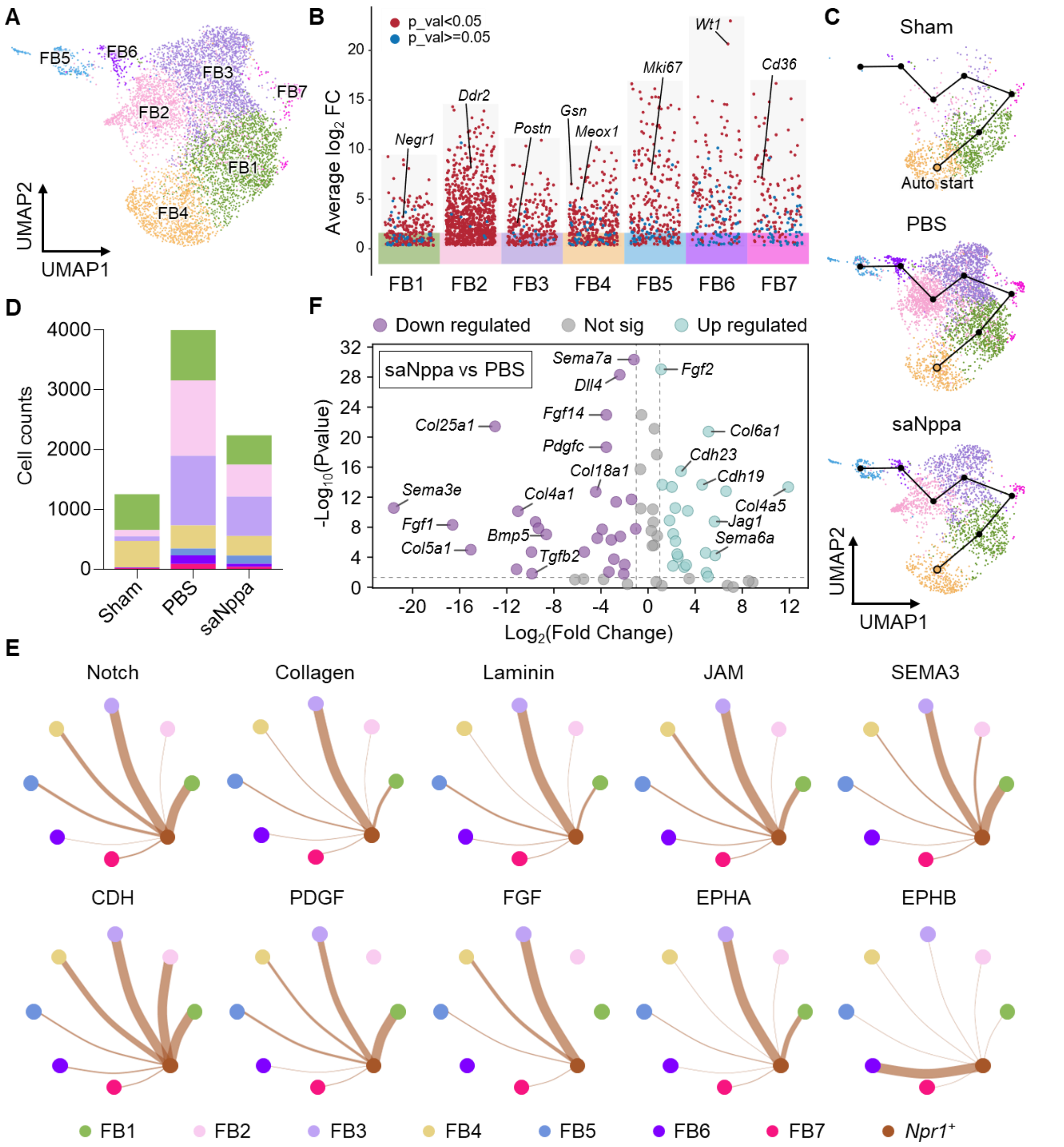

Fig. S14. Sub-clustering of fibroblasts.

(**A**) UMAP visualization of 7 FB subclusters. (**B**) Feature plots of marker genes for each FB subcluster. (**C**) Pseudotime trajectories of FB subclusters. (**D**) Cell population distribution of FB subclusters delineated into 3 groups and 7 subclusters. (**E**) Top 10 signaling pathways of cell-cell communication networks between *Npr1*$^+$ cells and FB subclusters. (**F**) Volcano plot showing differentially expressed ligands in *Npr1*$^+$ cells associated with intercellular communication between *Npr1*$^+$ cells and fibroblast subclusters.

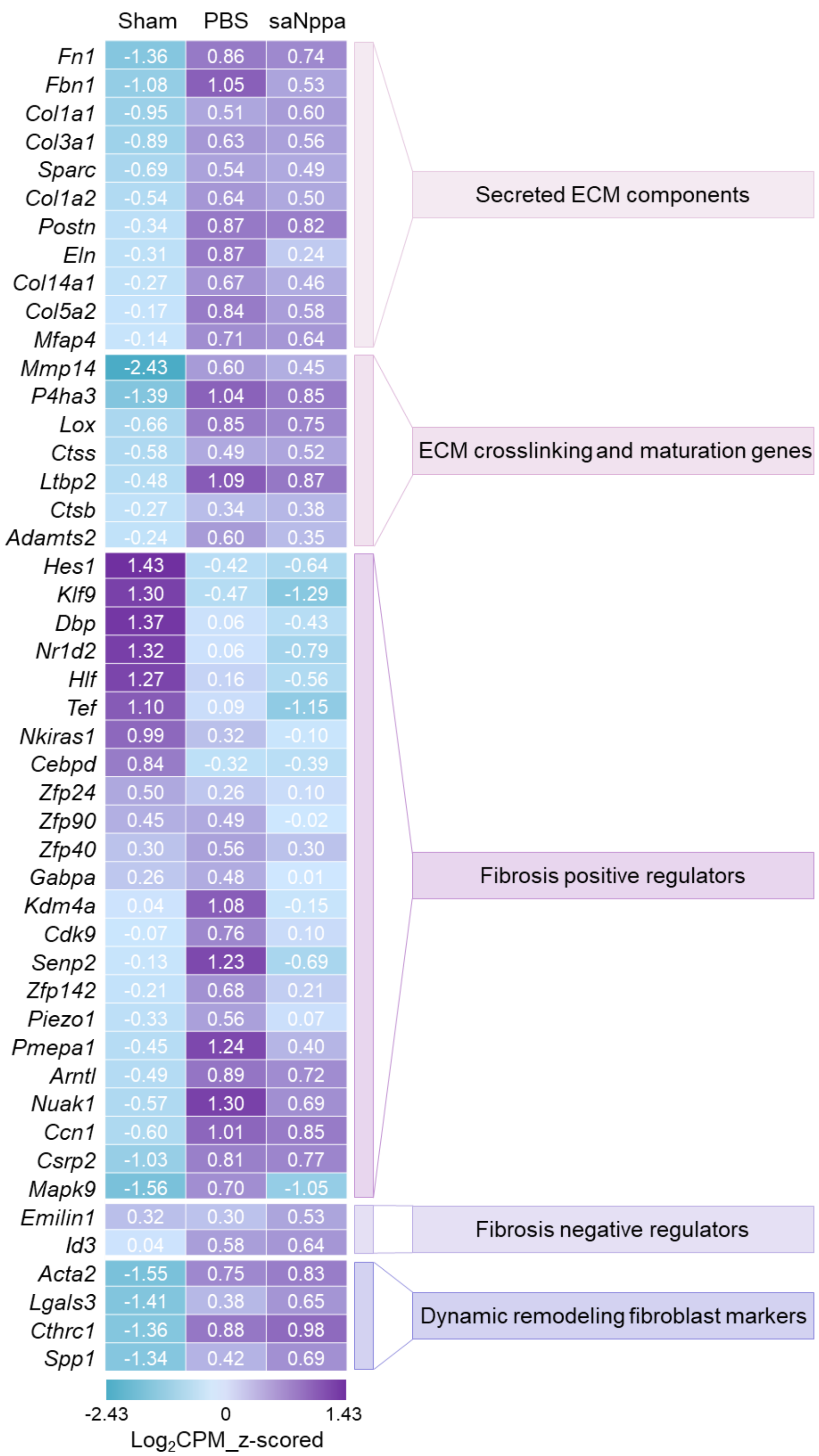

Fig. S15. Heatmap of pseudobulk fibrosis related gene expression in FB3 subcluster. Expression values are log_2 counts per million (log_2CPM) and row-scaled (*z*-score) across samples. The color bar represents *z*-scored log_2CPM values. Fibrosis-related genes are grouped into five functional categories.

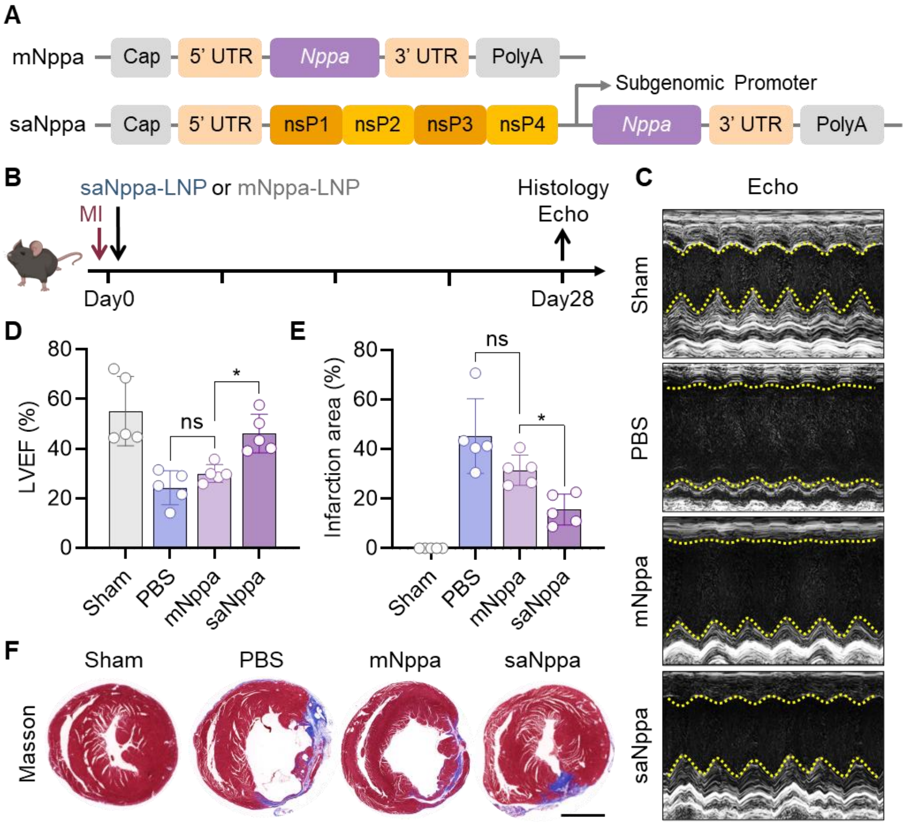

Fig. S16. Therapeutic potency of saNppa-LNPs versus mNppa-LNPs.

(**A**) Schematic illustrating the structure of *Nppa* self-amplifying RNA (saNppa) and conventional mRNA (mNppa). (**B**) Schematic representation of the experimental timeline. Mice underwent LAD ligation and received IM injection of 0.25 mg/kg saNppa-LNPs or mNppa-LNPs on the same day. Echocardiographic analysis and Masson's trichrome staining were performed on day 28 after MI. (**C**) Representative echocardiography images. (**D**) Quantification of LV ejection fraction. Data are presented as mean ± SD. n = 5. Statistical analysis was performed using one-way ANOVA with Tukey's multiple comparison test. $^*P < 0.05$ between the indicated groups. ns, not significant. (**E**) Quantification of infarction area from Masson's trichrome staining results. Data are presented as mean ± SD. n = 5. Statistical analysis was performed using one-way ANOVA with Tukey's multiple comparison test. $^*P < 0.05$ between the indicated groups. ns, not significant. (**F**) Representative Masson's trichrome staining images of whole heart cross sections. Scale bar, 2 mm.

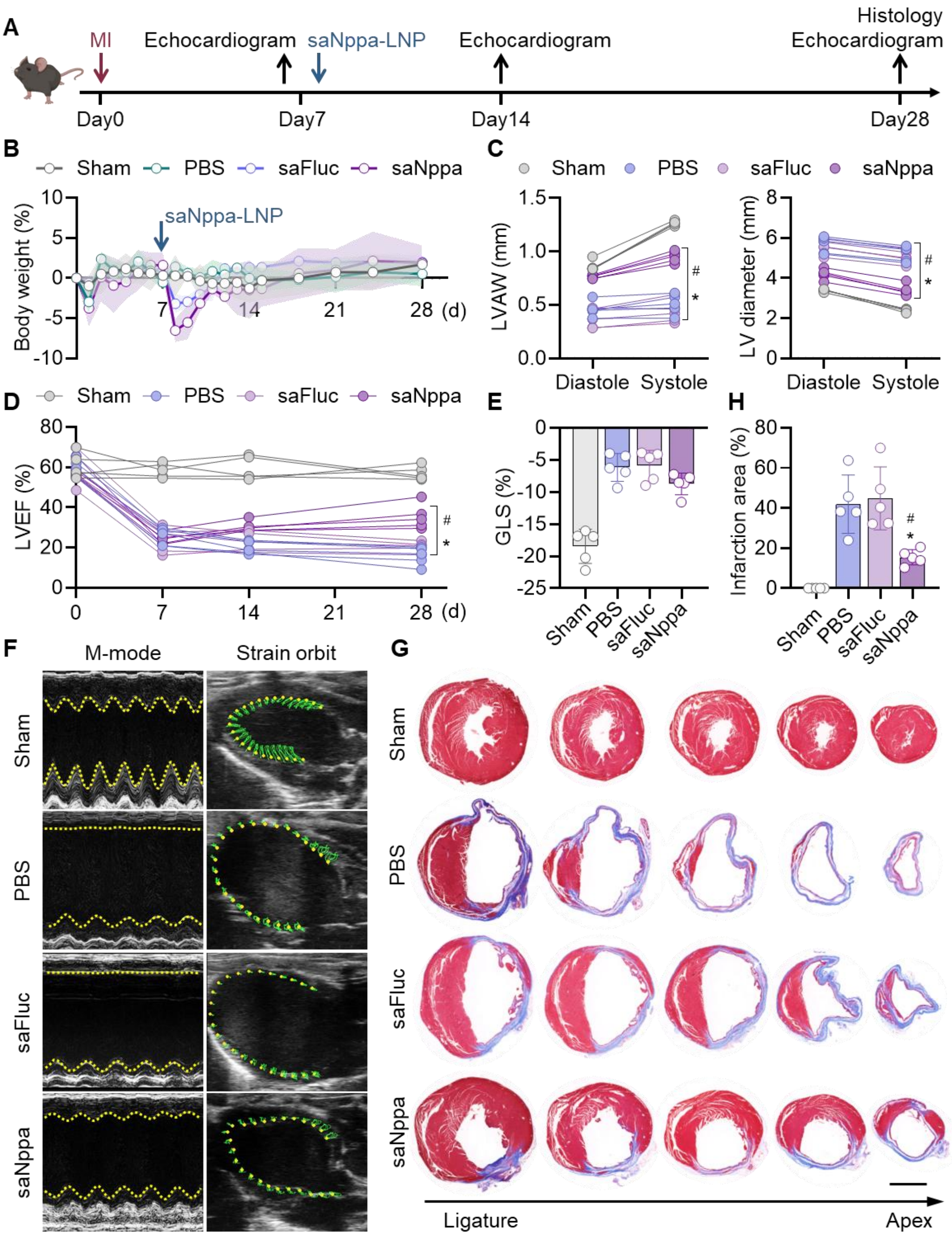

Fig. S17. Cardioprotective effects of delayed saNppa-LNP treatment.

(**A**) Schematic representation of the experimental timeline. Female mice underwent a LAD ligation on day 0 and received IM injection of 0.25 mg/kg saNppa-LNPs on day 7 after MI. Echocardiographic analysis was performed on day 7, 14, and 28 after MI. On day 28, heart

tissues were collected for histological examination. (**B**) Body weight changes after MI and a delayed saNppa-LNP treatment. Data are presented as mean ± SD. n = 5. (**C**) Measurements of LV anterolateral wall thickness and LV diameter. (**D**) LV ejection fraction on day 7, 14, and 28 after MI. Data are presented as individual values. n = 5. Statistical analysis was performed using two-way ANOVA with Tukey's multiple comparison test. $^{*}P < 0.05$ versus PBS, $^{\#}P < 0.05$ versus saFluc. (**E**) Quantification of LV global longitudinal strain. Data are presented as mean ± SD. n = 5. Statistical analysis was performed using one-way ANOVA with Tukey's multiple comparison test. (**F**) Representative echocardiography images and strain orbits. (**G**) Masson's trichrome staining images of whole heart cross sections. Scale bar, 2 mm. (**H**) Quantification of infarction area. Data are presented as mean ± SD. n = 5. Statistical analysis was performed using one-way ANOVA with Tukey's multiple comparison test. $^{*}P < 0.05$ versus PBS, $^{\#}P < 0.05$ versus saFluc.

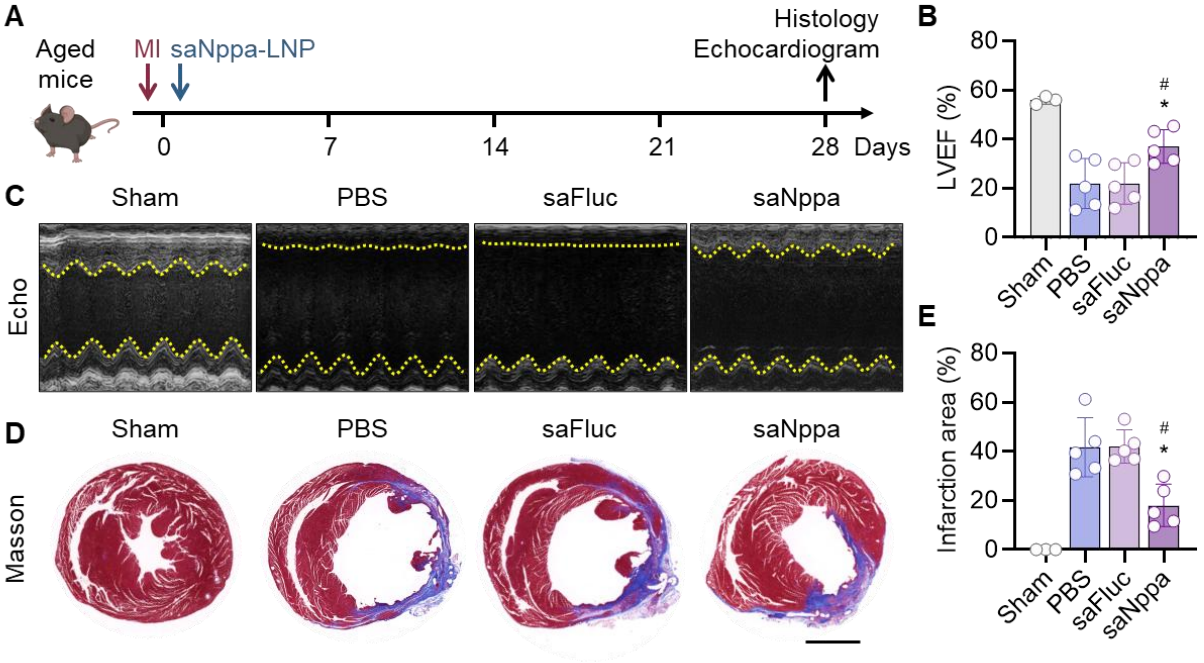

Fig. S18. Cardioprotective effects of saNppa-LNPs in aged mice.

(**A**) Schematic representation of the experimental timeline. 18-month-old mice were performed a LAD ligation on day 0 and intramuscularly injected 0.25 mg/kg saNppa-LNPs on the same day. Echocardiographic analysis and Masson's trichrome staining were performed on day 28 after MI. (**B**) Measurements of LV ejection fraction. Data are presented as mean ± SD. n = 3 or 5. Statistical analysis was performed using one-way ANOVA with Tukey's multiple comparison test. $^{*}P < 0.05$ versus PBS, $^{\#}P < 0.05$ versus saFluc. (**C**) Representative echocardiography images. (**D**) Masson's trichrome staining of whole heart cross sections. Scale bar, 2 mm. (**E**) Quantification of infarction area. Data are presented as mean ± SD. n = 3 or 5. Statistical analysis was performed using one-way ANOVA with Tukey's multiple comparison test. $^{*}P < 0.05$ versus PBS, $^{\#}P < 0.05$ versus saFluc.

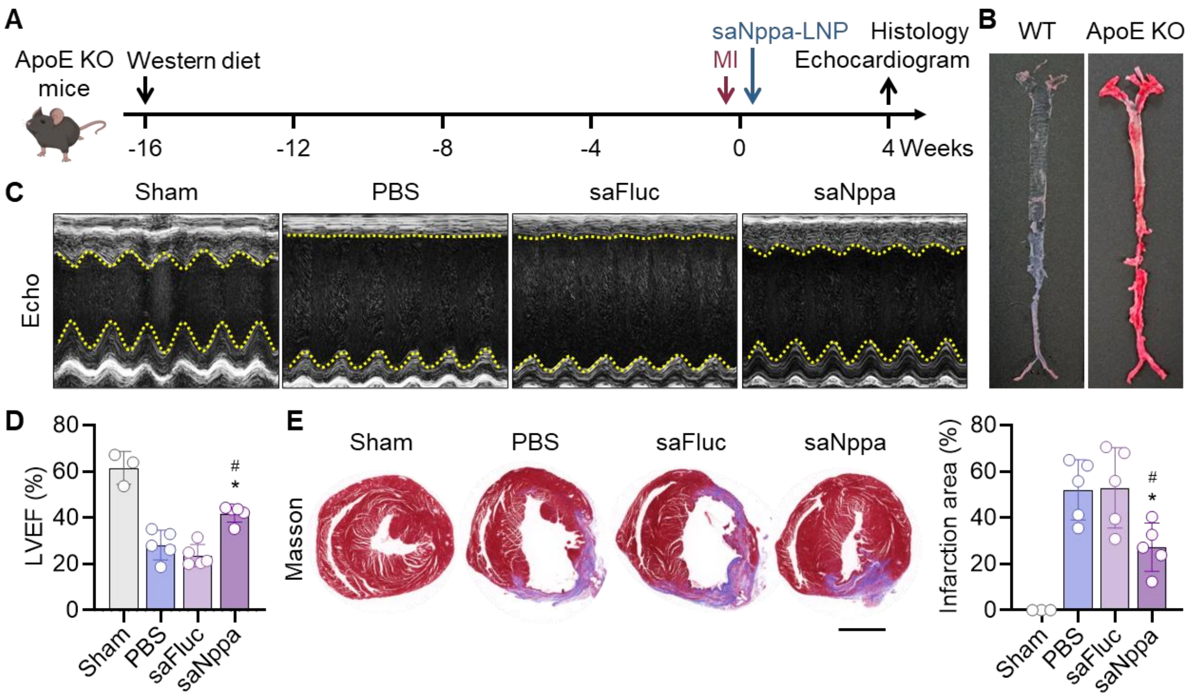

Fig. S19. Cardioprotective effects of saNppa-LNPs in an atherosclerosis MI model.

(**A**) Schematic representation of the experimental timeline. An atherosclerosis model was established by using *Apoe* knockout (ApoE KO) mice fed a Western diet for 16 weeks, after which MI was induced by LAD ligation and mice received IM injection of 0.25 mg/kg saNppa-LNPs. Echocardiographic analysis and Masson's trichrome staining were performed on day 28 after MI. (**B**) Representative images of oil red O staining of aorta from normal and atherosclerosis mouse. (**C**) Representative echocardiography images. (**D**) Measurements of LV ejection fraction. Data are presented as mean ± SD. n = 3 or 5. Statistical analysis was performed using one-way ANOVA with Tukey's multiple comparison test. $^{*}P < 0.05$ versus PBS, $^{\#}P < 0.05$ versus saFluc. (**E**) Masson's trichrome staining of whole heart cross sections and infarction area quantification. Data are presented as mean ± SD. n = 3 or 5. Statistical analysis was performed using one-way ANOVA with Tukey's multiple comparison test. $^{*}P < 0.05$ versus PBS, $^{\#}P < 0.05$ versus saFluc. Scale bar, 2 mm.

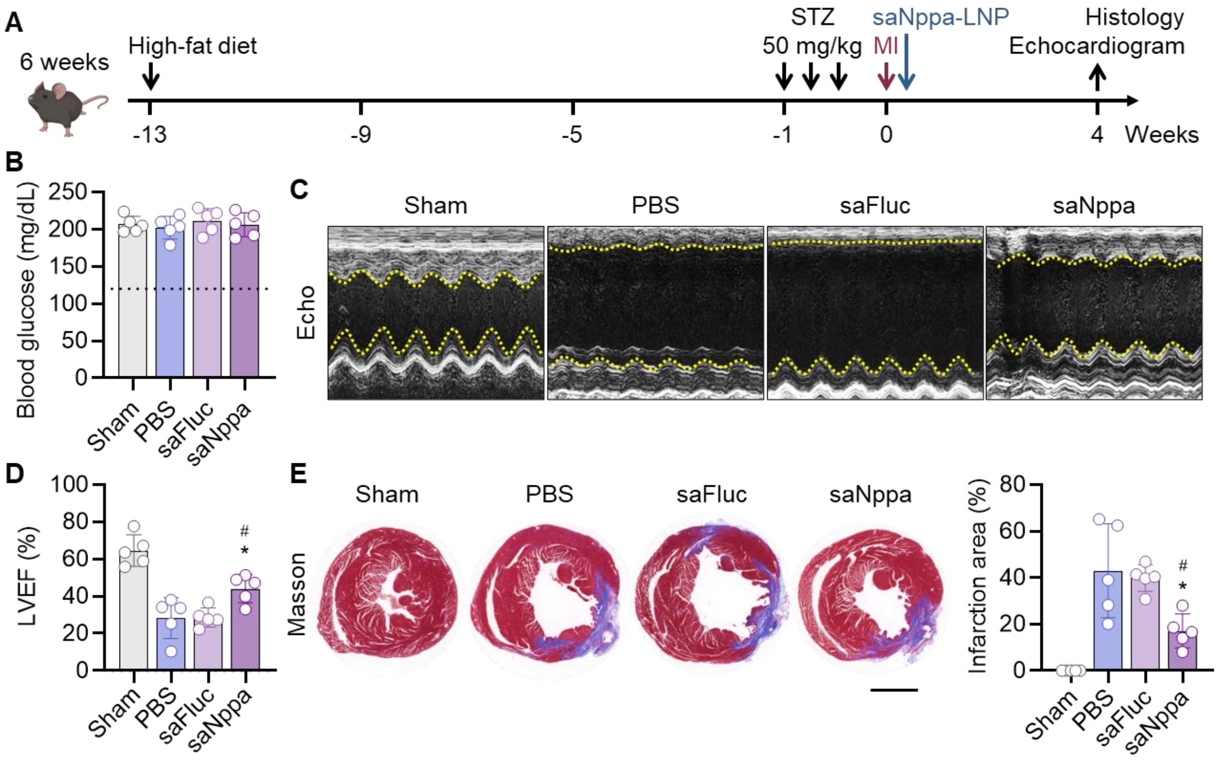

Fig. S20. Cardioprotective effects of saNppa-LNPs in a metabolic syndrome MI model.
(**A**) Schematic representation of the experimental timeline. A metabolic syndrome model generated by high-fat diet plus 3 injections of streptozotocin (STZ, 50 mg/kg) to mimic type 2 diabetes. After confirming that blood glucose elevated to ~ 200 mg/dL, LAD ligation induced MI and IM injection of 0.25 mg/kg saNppa-LNPs were performed on the same day. Echocardiographic analysis and Masson's trichrome staining were performed on day 28 after MI. (**B**) Blood glucose levels before MI. Data are presented as mean ± SD. n = 5. (**C**) Representative echocardiography images. (**D**) Measurements of LV ejection fraction. Data are presented as mean ± SD. n = 5. Statistical analysis was performed using one-way ANOVA with Tukey's multiple comparison test. *$P < 0.05$ versus PBS, #$P < 0.05$ versus saFluc. (**E**) Masson's trichrome staining of whole heart cross sections and infarction area quantification. Data are presented as mean ± SD. n = 5. Statistical analysis was performed using one-way ANOVA with Tukey's multiple comparison test. *$P < 0.05$ versus PBS, #$P < 0.05$ versus saFluc. Scale bar, 2 mm.

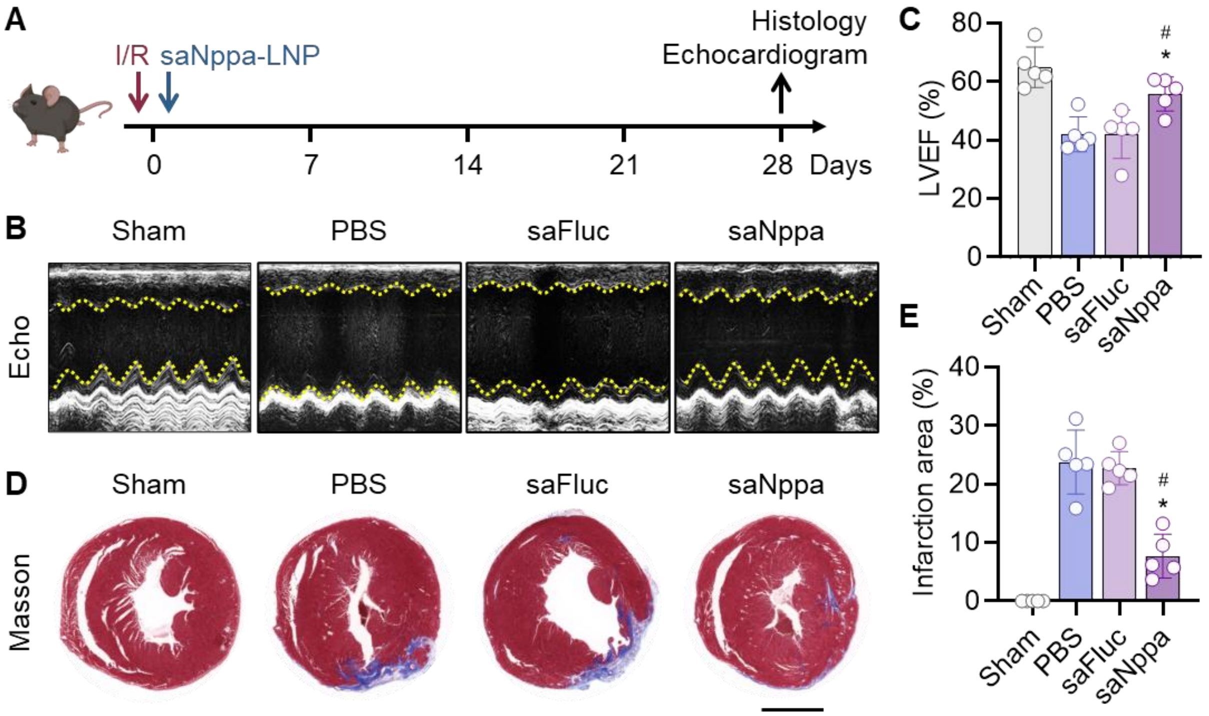

Fig. S21. Cardioprotective effects of saNppa-LNPs in an I/R model.

(**A**) Schematic representation of the experimental timeline. Mice were performed a LAD ligation for 45 min and then reperfusion. 0.25 mg/kg saNppa-LNPs were intramuscularly injected on the same day. Echocardiographic analysis and Masson's trichrome staining were performed on day 28 after MI. (**B**) Representative echocardiography images. (**C**) Measurements of LV ejection fraction. Data are presented as mean ± SD. n = 5. Statistical analysis was performed using one-way ANOVA with Tukey's multiple comparison test. **P* < 0.05 versus PBS, #*P* < 0.05 versus saFluc. (**D**) Masson's trichrome staining of whole heart cross sections. (**E**) Quantification of infarction area. Data are presented as mean ± SD. n = 5. Statistical analysis was performed using one-way ANOVA with Tukey's multiple comparison test. **P* < 0.05 versus PBS, #*P* < 0.05 versus saFluc. Scale bar, 2 mm.

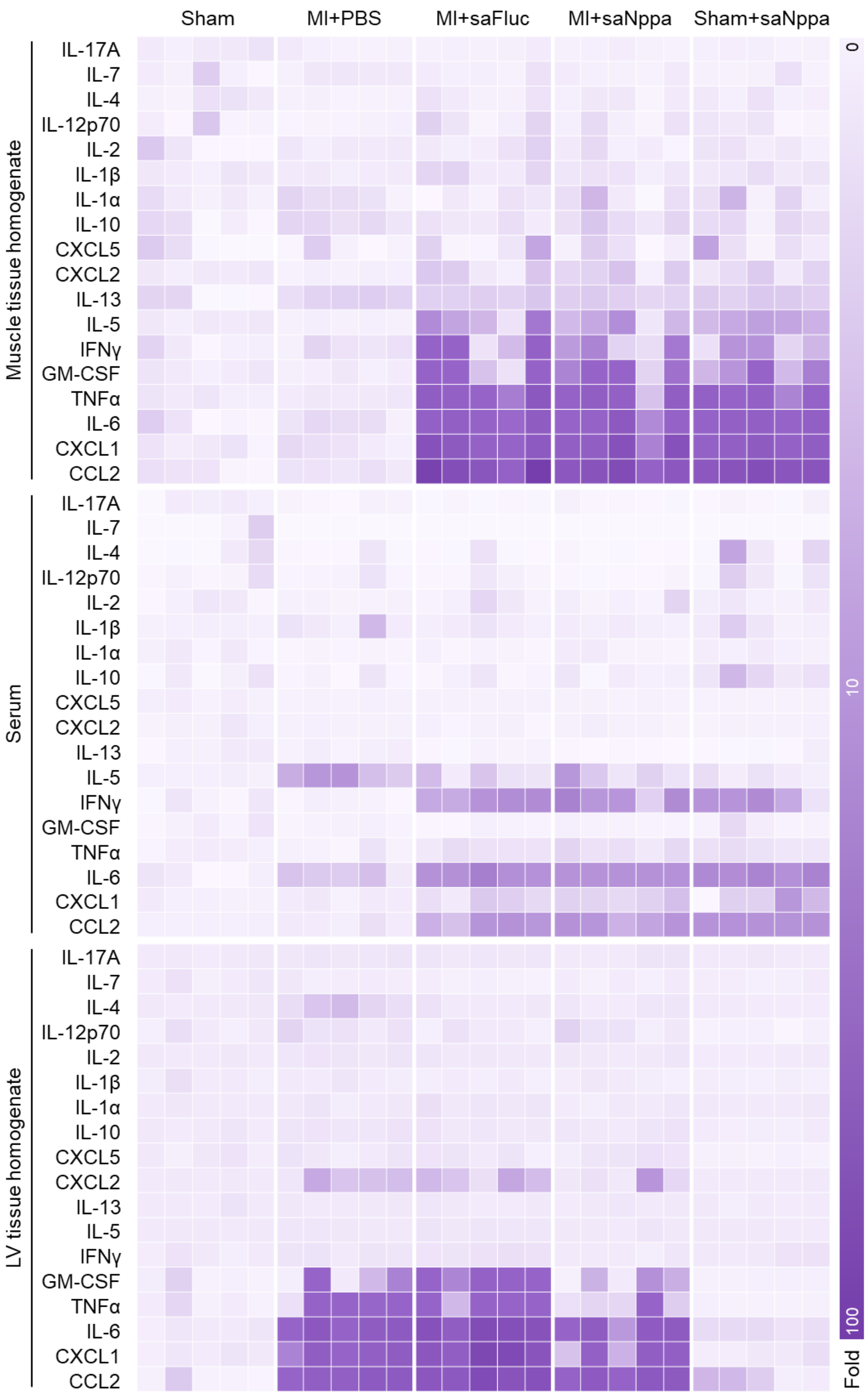

Fig. S22. Inflammatory response after saNppa-LNP injection.

Heatmap showing concentration of 18 inflammatory cytokines in the injected muscle tissue homogenates, sera, and LV tissue homogenates within 24 hours after IM injection of 0.25 mg/kg saFluc-LNPs or saNppa-LNPs. Data were normalized to the mean value of samples collected from sham-operated mice.

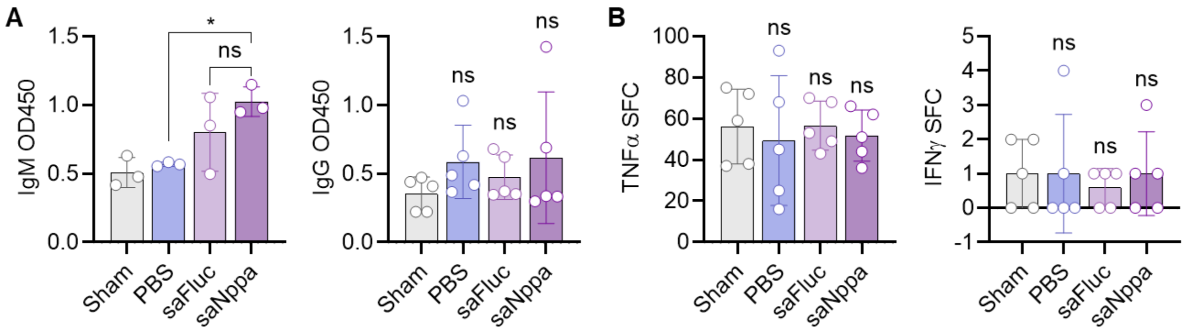

Fig. S23. Humoral and cellular immune responses after saNppa-LNP injection.

(**A**) IgM and IgG levels from serum collected on day 3 and day 28 respectively after MI injection of saNppa-LNPs. Data are presented as mean ± SD. n = 3 or 5. Statistical analysis was performed using one-way ANOVA with Tukey's multiple comparison test. $*P < 0.05$ between the indicated groups. ns, not significant. (**B**) Enzyme-linked immunospot (ELISpot) assay of TNFα and IFNγ producing spot-forming cells (SFC) after saNppa-LNP treatment. Data are presented as mean ± SD. n = 5. Statistical analysis was performed using one-way ANOVA with Tukey's multiple comparison test. ns, not significant.

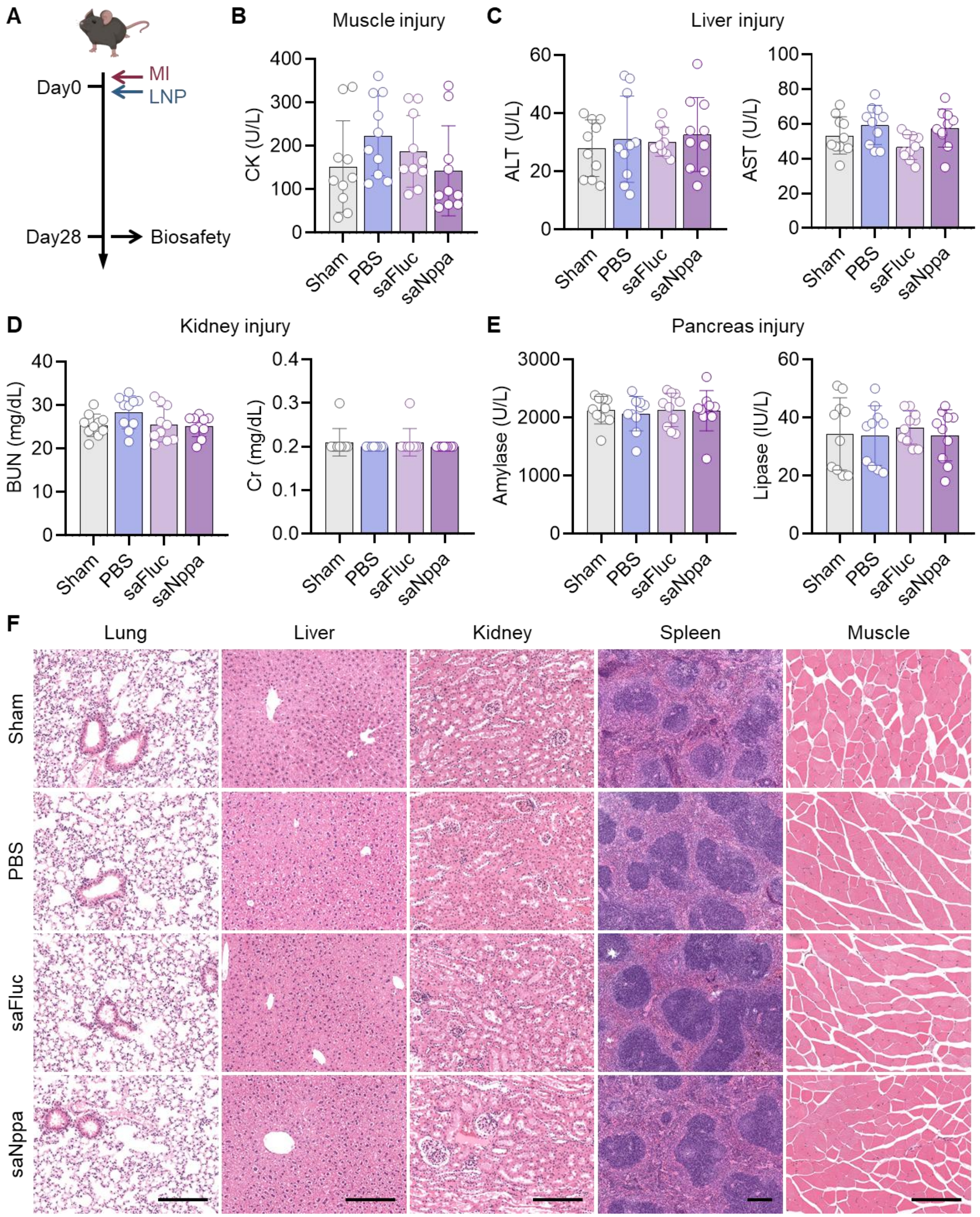

Fig. S24. Biosafety verification of saNppa-LNP treatment in mice.

(**A**) Timeline of biosafety verification in this study. LAD ligation and saNppa-LNP injection (0.25 mg/kg) were performed on day 0. Blood and tissue samples were collected 28 days later. (**B-E**) Chemistry panel tests of (**B**) muscle injury indicator (creatine kinase, CK), (**C**) liver injury indicators (alanine transaminase and aspartate aminotransferase, ALT and AST), (**D**) kidney injury indicators (blood urea nitrogen and creatinine, BUN and Cr), and (**E**) pancreas injury

indicators (amylase and lipase) on day 28 after MI and saNppa-LNP injection. Data are presented as mean ± SD. n = 10. Both female and male mice serum samples were included here. Statistical analysis was performed using one-way ANOVA with Tukey's multiple comparison test. (**F**) H&E staining of main organs (lung, kidney, spleen and liver) and injected muscle tissues after 28 days after MI and saNppa-LNP injection. Scale bars, 200 μm.

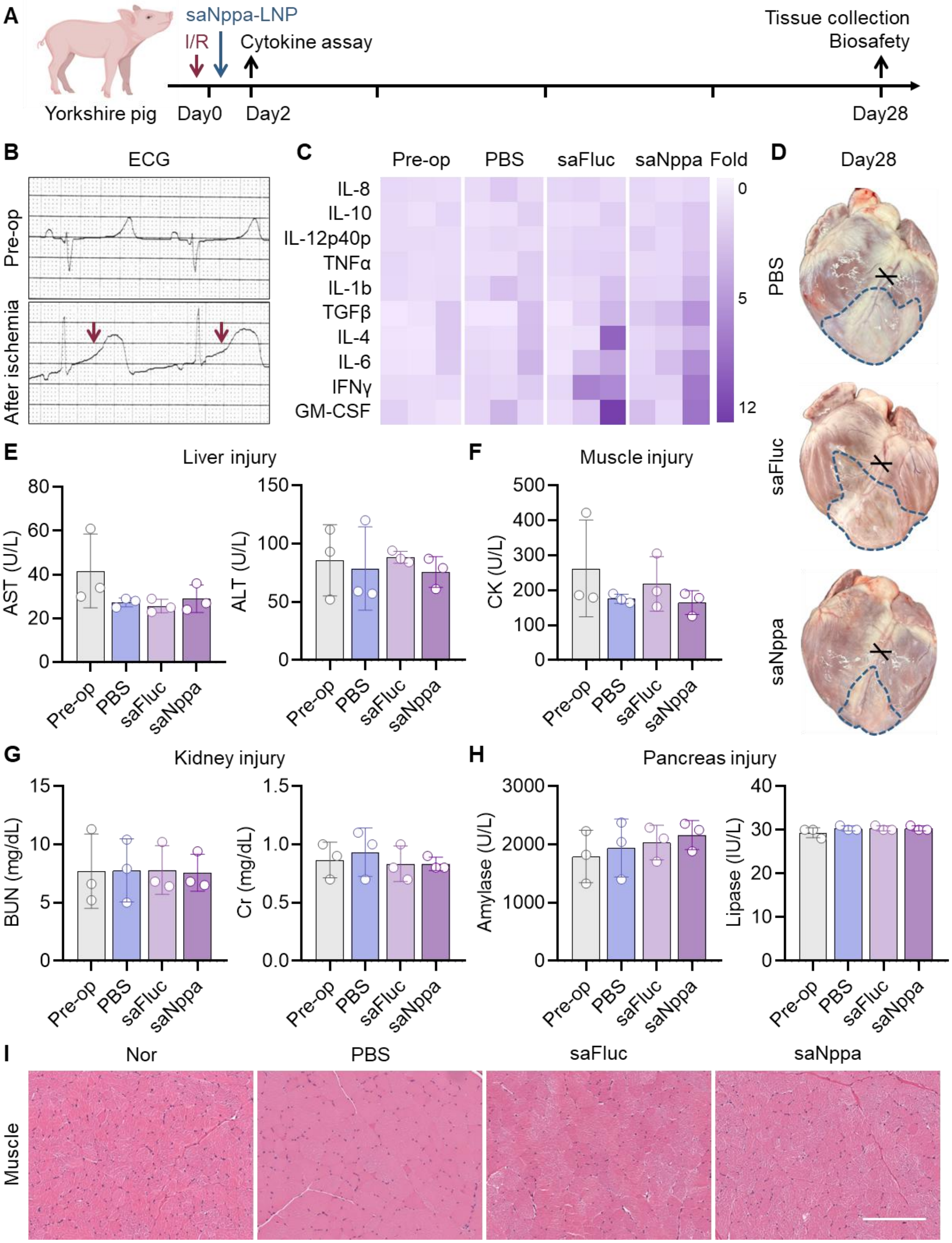

Fig. S25. Efficacy and safety of saNppa-LNP therapy in a swine model of I/R injury.

(**A**) Timeline of the biosafety verification in this study. A swine I/R model was induced in Yorkshire pigs by balloon occlusion intervention for 90 minutes followed by reperfusion. During recovery, 20 μg/kg saNppa-LNPs were intramuscularly injected. Sera were collected on day 2

for cytokine assay. Hearts and injected muscle tissues were collected on day 28 after MI. (**B**) Representative electrocardiogram (ECG) during ischemia in the surgery. Arrows point to the ST-segment elevation. (**C**) Heatmap showing concentrations of 10 inflammatory cytokines from sera on day 2 after saFluc-LNP or saNppa-LNP injection. Data were normalized to the mean value of the samples collected before operation. (**D**) Representative images of the heart on day 28. Fibrosis regions are circled by the blue dashed lines. (**E-H**) Chemistry panel tests of (**E**) liver injury indicators (alanine transaminase and aspartate aminotransferase, ALT and AST), (**F**) muscle injury indicators (creatine kinase, CK), (**G**) kidney injury indicators (blood urea nitrogen and creatinine, BUN and Cr), and (**H**) pancreas injury indicators (amylase and lipase) on day 28 after MI and injections. Data are presented as mean ± SD. n = 3. (**I**) H&E staining of injected muscle tissues on day 28 after saNppa-LNP injection. Scale bar, 200 μm.

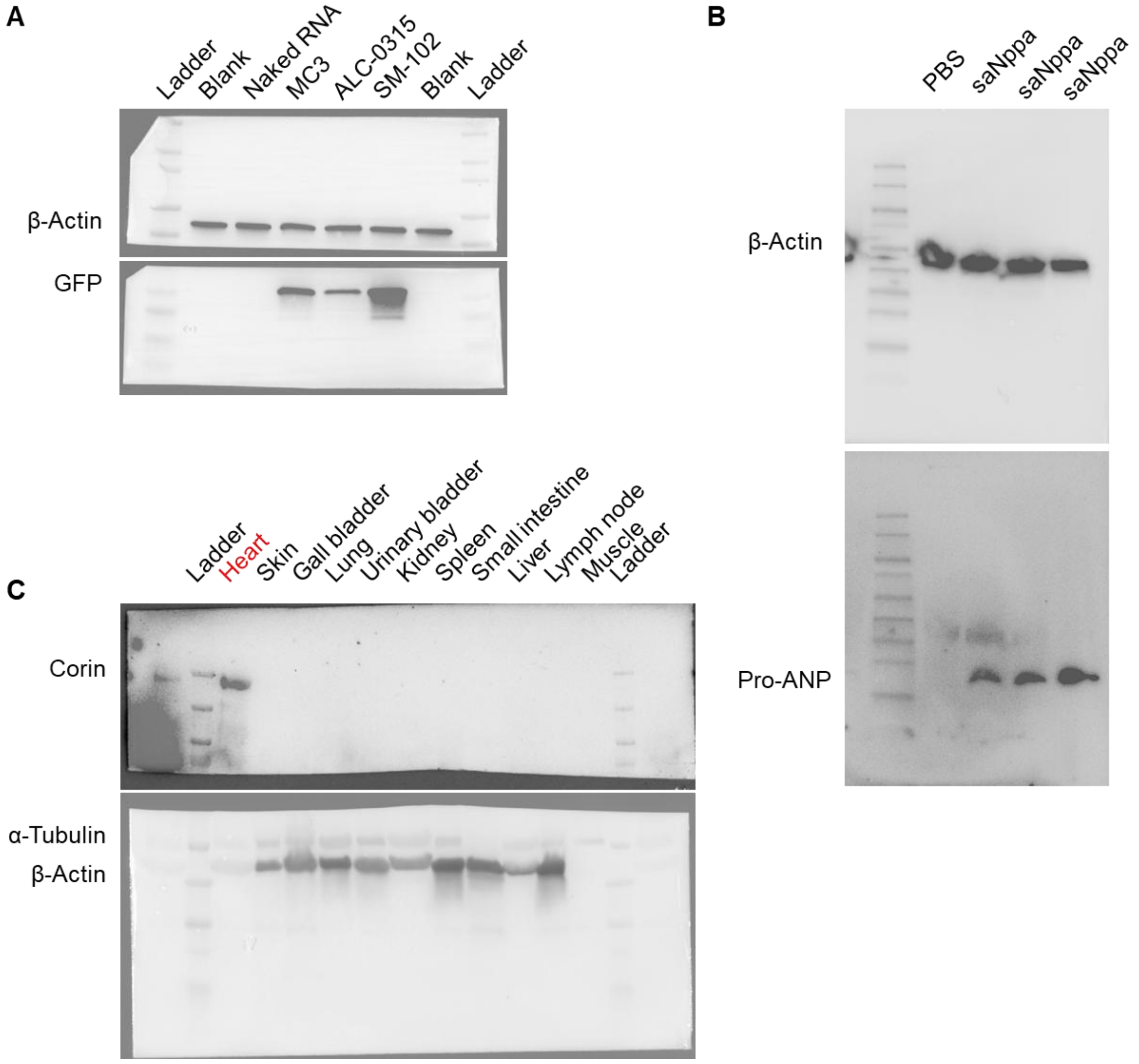

Fig. S26. Original images of western blot results.

(**A**) Original images of fig. S2F. (**B**) Original images of fig. S4B. (**C**) Original images of fig. S6E.

Table S1. Formulation of LNPs used in this study.

Formulations	LNP-1	LNP-2	LNP-3
Ionizable cationic lipid	DLin-MC3-DMA	ALC-0315	SM-102
Neutral phospholipid	DSPC	DSPC	DSPC
Sterol lipid	Cholesterol	Cholesterol	Cholesterol
PEGylated lipid	DMG-PEG (2000)	ALC-0159	DMG-PEG (2000)
Lipid molar ratio	50:10:38.5:1.5	46.3:9.4:42.7:1.6	50:10:38.5:1.5

LNPs were formulated using lipid components employed in FDA-approved LNP formulations. The table summarizes the compositions and lipid molar ratios of the three formulations evaluated in this study. Lipid molar ratios were shown as ionizable cationic lipid: neutral phospholipid: sterol lipid: PEGylated lipid.
